# Selective targeting of kinesin on lipid droplets in the liver reduces plasma lipids

**DOI:** 10.1073/pnas.2528332123

**Published:** 2026-05-12

**Authors:** Subham Kumar Tripathy, Archisman Mahapatra, Ojal Saharan, Hindol Chatterjee, Neelanjana Sengupta, Siddhesh Kamat, Sreelaja Nair, Roop Mallik

**Affiliations:** ^a^ Department of Biosciences and Bioengineering, Indian Institute of Technology Bombay, Mumbai 400076, India; ^b^ Department of Biology, Indian Institute of Science Education and Research, Pune 411008, India; ^c^ Department of Biological Sciences, Indian Institute of Science Education and Research Kolkata, Mohanpur 741246, India

**Keywords:** lipid droplets, lipoproteins, VLDL, hyperlipidaemia, kinesin

## Abstract

The liver controls plasma lipids by secreting lipid-rich very low density lipoproteins (VLDL) into blood. Inside hepatocytes in the liver, Lipid Droplets (LDs) are transported to the Smooth Endoplasmic Reticulum by kinesin-1 motors, where they are catabolized to supply lipids for VLDL assembly. Here we find that kinesin-1 uses its tail domain to bind the monolayer phospholipid membrane of LDs, but alternative mechanisms to bind cellular organelles with bilayer membranes. A peptide corresponding to the tail domain of kinesin-1 therefore competes with and removes kinesin-1 selectively from LDs with minimal effect on other organelles. Delivery of lipids for VLDL assembly is consequently reduced, causing a remarkable reduction of ~50% of secreted lipids (triglycerides and cholesterol) in cell culture. Strikingly, the peptide causes no unwanted accumulation of lipids inside cells because it redistributes LDs across the cell, enhancing LD-to-mitochondria lipid trafficking for mitochondrial lipid utilization. Further, we show that egg-liposomes can be used to orally deliver the kinesin tail domain peptide to zebrafish. The peptide accumulates in the zebrafish liver, and reverses diet-induced hyperlipidemia to bring zebrafish back to a normolipidemic state. Reflecting its effects in cell culture, the peptide causes no unwanted hepatic accumulation of lipids, no toxicity, and no developmental or behavioral defects in zebrafish. Using a peptide to displace proteins (e.g., kinesin) selectively from LDs provides a radically different approach against lipid disorders. This monolayer-vs.-bilayer strategy can potentially be extended to target other LD-bound proteins that function as key regulators of Lipid metabolism.

## Significance

We show that a peptide corresponding to the Kinesin Tail Domain selectively removes kinesin-1 motors from Lipid Droplets (LDs), thus blocking the delivery of lipids for very low density lipoproteins (VLDL) assembly inside hepatocytes in the liver. We demonstrate that kinesin-1 binds to LDs using its tail domain, but to other cellular organelles using alternative mechanisms. Thus, the peptide removes kinesin-1 only from LDs with no observable effect on other membrane trafficking pathways. We develop a method to deliver this peptide to the liver of zebrafish larvae and adults using Egg Liposomes, causing a remarkable reduction of Serum Triglycerides and Cholesterol. This ability to manipulate lipid trafficking selectively from LDs could serve as a potentially novel route to target hyperlipidemia.

**Fig. 1.**
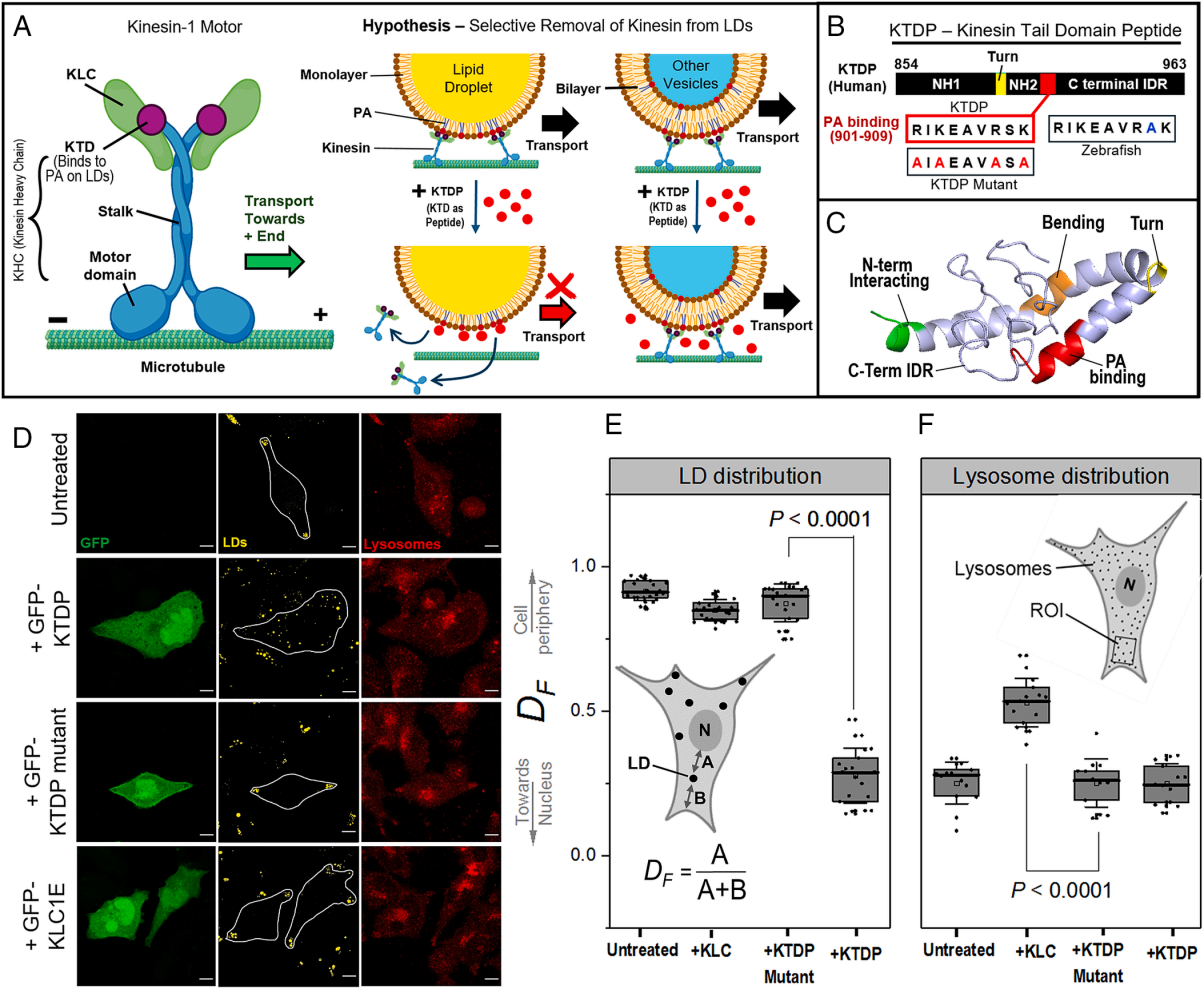
Selective targeting of LD transport using a KTDP. (*A*) Cartoon of the dimeric Kinesin-1 (Conventional Kinesin) Motor. KHC gene codes for the N-terminal Motor domain, Coiled coil stalk, and C-terminal KTD. KLC is expressed by the KLC gene. Two KHC and two KLC units combine to assemble the dimeric motor. Hypothesis depicts that kinesin-1 binds to LDs through its tail domain, thus a KTDP can compete with and remove kinesin-1 from LDs. KTDP has minimal effect on other (bilayer) organelles which use alternative mechanisms (e.g., the KLC) to bind kinesin-1. Thus, LD transport can be targeted selectively to reduce delivery of lipids for lipoprotein assembly in hepatocytes. (*B*) Details of human KTDP showing predicted N-terminal helices (NH1 and NH2), “turn” region, and a C-terminal intrinsically disordered region (C-Term IDR). The PA binding domain and its AA sequence are also shown. Mutations in basic AAs that block PA-binding of KTD (relevant to the KTDP-Mutant) are also shown. KTD sequence of PA-binding domain in zebrafish is also shown. (*C*) Predicted secondary structure of KTDP obtained from modeling in I-TASSER. (*D*) Confocal images of McARH-7777 hepatoma cells (Untreated Control; Overexpressing GFP-KTDP, GFP-KTDP-mutant, and GFP-KLC1E constructs). LDs (Yellow pseudocolor: stained by MDH dye) and lysosomes (Red: stained using LAMP1 antibody) are shown in separate channels. Some cell outlines are marked in the LD channel. (Scale bar, 10 µm.) (*E*) Quantification of effects of overexpressing KTDP and KLC peptides on LDs using the parameter *D_F_* (see figure for definition). *D_F_* = 1 and *D_F_* = 0 respectively imply peripheral and perinuclear localization of LDs. Data represent Mean ± SD. (*F*) Effects of overexpressing KTDP and KLC peptides on lysosome distribution. Lysosomes are quantified by measuring LAMP1 intensity within regions of interest near the cell periphery, and compared to LAMP1 intensity at whole-cell level. Ratio of peripheral to whole-cell LAMP1 intensity is then calculated (see main text). Each data point represents the distribution from an independent cell. Data are Mean ± SD.

Lipids are stored inside cells in the form of triacylglycerol (TG) and Cholesterol esters (CE) that make up the neutral-lipid core of lipid droplets (LDs). LDs also harbor lipid-synthesizing enzymes, lipases, and vesicular transport motors revealing LDs as dynamic hubs where lipids are stored or consumed to channel energy for metabolic demands (1–4). LDs share many common phospholipids and proteins with the ER (5, 6), however, LDs are different in that their bounding membrane is a phospholipid monolayer (Fig. 1*A*). This monolayer membrane has unique biophysical properties (2), causing specific proteins to be targeted to LDs in response to metabolic and immunological cues (7, 8). Unlike other organelles, no specific protein-targeting machinery has been identified for LDs. Rather, proteins could bind LDs via packing defects that appear on the LD membrane when TG is transiently exposed to the cytosol (9). LD proteins may thus belong to two classes (10, 11). Class I proteins translocate from the ER to LDs, and bind LDs through a hairpin structure (e.g., GPAT4, DGAT2). Class II proteins translocate from cytosol to LDs and often form amphipathic helices upon interaction with LDs (e.g., perilipins, CCT1).

Because LD-bound proteins hold the key for living organisms to access their energy/lipid reserves, their function is widely discussed in the context of LD biogenesis, catabolism, and metabolic disorders (10–12). Surprisingly, we find no discussion of the possibility that a protein’s presence on LDs could be manipulated against precisely these metabolic disorders. This is pertinent, considering the rising worldwide epidemic of Hyperlipidemia and Obesity with very limited options available against these conditions (13–15). With this background, and because LDs are the only cytosolic organelle bounded by a monolayer membrane, we made the following HYPOTHESIS (Fig. 1*A*):- Suppose that a given protein binds to monolayer or bilayer membranes using two different domains. A peptide that mimics the monolayer-binding domain of this protein should then compete with, and remove the protein from LDs with higher efficiency as compared to bilayer vesicles. This peptide could therefore inhibit LD-specific pathways downstream to that LD-resident protein with minimal interference in cellular functions of the same protein on bilayer membranes. To the best of our knowledge, such a possibility has never been discussed or demonstrated in the context of targeting LD biology and metabolic disorders.

Here we will test the above hypothesis for targeting the kinesin-1 motor’s attachment to LDs vs. other (non-LD) bilayer vesicles. Kinesin-1 is composed of two kinesin heavy chains (KHCs) and two light chains (KLCs) that are encoded by different genes and assembled into a dimeric motor (Fig. 1*A*). The KHC consists of a motor, a coiled-coil stalk, and a C-terminal kinesin tail domain (KTD) (16, 17). Kinesin-1 is recruited to many kinds of bilayer vesicles via protein complexes (e.g., Miro-Milton) and/or adaptors that interact with the KLC (18, 19). Kinesin-1 also binds to and transports LDs in *Drosophila* embryos (20) and in the rat liver (21, 22). We showed that insulin signaling activates phospholipase-D inside the liver to generate phosphatidic acid (PA) on LDs inside hepatocytes. Kinesin-1 then binds directly to PA, causing LD transport to the smooth-ER located at the periphery of hepatocytes (7, 22). At the smooth-ER, these LDs supply ~70% of the lipid content of VLDL particles (23–25). Kinesin-1 knockdown reduced TG secretion from cells and also lowered serum-TG in animals (rats) because kinesin mediated delivery of TG-rich LDs for VLDL assembly was reduced (22). Indeed, the peak of ApoB-100 (a VLDL marker) was shifted to higher density in sucrose gradient fractions, showing that TG-deficient VLDL particles of higher density were circulating in the blood of rats after kinesin-1 knockdown (22).

While kinesin-1 knockdown did reduce serum-TG in animals, this cannot be a viable strategy to control plasma lipids because kinesin-1 has many other essential functions inside cells (18, 19). However, we found later that overexpressing the C-terminal KTD (aa 854-963 of KHC—see Fig. 1 *B* and *C*) as a peptide disrupted only the peripheral distribution of LDs inside cells without affecting localization of lysosomes, early endosomes, and mitochondria (7). To explain these findings, here we hypothesize that kinesin-1 binds to LDs via the KTD (Fig. 1*A*). Thus, the KTD peptide (hereafter KTDP) can competitively displace endogenous kinesin-1 from LDs to disrupt LD transport and reduce the lipid content inside VLDL particles. If KTDP is less effective in removing kinesin from other (bilayer) cargoes of kinesin, then cellular functions outside the LD-VLDL axis should not be perturbed significantly by KTDP. We support this hypothesis using in vitro assays, MD simulations, and cell culture experiments. We further demonstrate that liposomes prepared from eggs (26) are novel and facile in-vivo peptide delivery system to the liver of zebrafish larvae and adults. KTDP is delivered intact to the liver, causing a marked reduction in serum lipids (TG and cholesterol) in the zebrafish model which is widely used for studying lipid metabolism (27, 28). KTDP causes no adverse lipid accumulation in cell culture or in larvae, which continue to develop and behave normally. This strategy to reduce serum lipids by inhibiting kinesin-mediated delivery of LDs for VLDL assembly in the liver could provide a radically different approach against the growing epidemic of hyperlipidemia.

## Results

### The KTD Peptide Specifically Disrupts Peripheral Distribution of LDs Inside Cells.

We first tested the effect of overexpressing GFP-tagged KTDP on LD distribution in McA-RH7777 rat hepatoma cells which are known to secrete VLDL (7, 22, 29). We will use KTDP corresponding to the human KTD sequence throughout this work. *SI Appendix*, Fig. S1*A* (*Left*) shows sequence alignment of KTD across the species relevant here (Human, Rat, Zebrafish). The helix region of KTD (aa 854 to 913; *Right* panel in *SI Appendix*, Fig. S1*A*) that is implicated in membrane binding (7, 30) is highly conserved, justifying the use of human KTDP in all our experiments. McA-RH7777 cells have an elongated morphology (Fig. 1*D*), with the smooth-ER located at the cell periphery (*SI Appendix*, Fig. S1*B*), where the plus-ends of microtubules (MTs) are present (7). This is strikingly similar to the peripheral localization of the smooth-ER seen in rat liver sections (7). Control (untreated) cells had most LDs at the cell periphery (Fig. 1*D*), suggesting high kinesin activity on LDs. Overexpressing GFP-tagged KTDP (GFP-KTDP) caused LDs to redistribute throughout the cells, but a mutant GFP-KTDP (GFP-KTDP-mut; Fig. 1*B*) that cannot bind to PA on LDs (7) showed no effect on LD distribution (Fig. 1*D*). We verified that GFP-KTDP and GFP-KTDP-mut are expressed at equal levels in cells (*SI Appendix*, Fig. S1*C*), as also found earlier for these plasmids (7). As described earlier (7), we quantified LD position using a fractional distance parameter (*D_F_*; Fig. 1*E*). *D_F_* = 1 and *D_F_* = 0 respectively indicate LDs at cell periphery and cell-center. GFP-KTDP treated cells had lower values of *D_F_* for LDs, but GFP-KTDP-mut caused no reduction in *D_F_* (Fig. 1*E*). Importantly, GFP-KTDP had no visual or quantifiable effect on lysosome distribution in McA-RH7777 cells (Fig. 1 *D* and *F*). We have shown that KTDP also has no effect on the distribution of mitochondria and early endosomes in these cells (7). In contrast to KTDP, overexpressing GFP tagged kinesin light chain (GFP-KLC1E) had no effect on LD distribution (Fig. 1*E*) but caused a peripheral distribution of lysosomes (Fig. 1*F*), as also reported earlier (19).

Based on these observations, KTDP has a very pronounced and selective effect on kinesin-driven transport of LDs toward the peripheral region of cells. This selectivity can be explained if kinesin-1 binds to LDs using its KTD, but uses other domains to bind bilayer organelles (e.g., KLC for lysosomes). In-vitro studies show that the KTD binds to kinesin’s motor domain and also to the microtubule. KTD inhibits the ATPase activity to switch kinesin into an “off state” (31, 32). It is therefore intriguing that McA-RH7777 cells stably overexpressing KTDP show no phenotype and grow normally, with no effect on the localization of lysosomes, endosomes, and mitochondria inside these cells (7). Why does the overexpressed KTDP not bind to the motor domain of kinesin-1 on bilayer organelles, and cause a general shutdown of kinesin-1 activity? This can be explained if association with KLC on bilayer organelles keeps kinesin-1 in an activated state that is immune to KTDP (32). We will return to these issues in *Discussion* section.

### In-Vitro Affinity of KTD Peptide to Monolayer and Bilayer Membranes.

The above data suggest that KTDP inhibits kinesin-1 on LDs, but is unable (or less able) to inhibit kinesin function on other vesicles. The simplest explanation for this observation is that overexpressed KTDP competes with the KTD of endogenous kinesin-1 for binding to LDs, and thus removes kinesin-1 from LDs. However, KTDP neither removes nor does it block kinesin-1 function on other (bilayer) vesicles because i) KTDP has lower affinity to bilayer membranes ii) Kinesin-1 does not bind to bilayer membranes using KTD (Hypothesis; Fig. 1*A*) and iii) KTDP does not inhibit ATPase activity of kinesin-1 that is bound to bilayer vesicles because KLC keeps kinesin-1 in an activated state that is immune to KTDP.

**Fig. 2.**
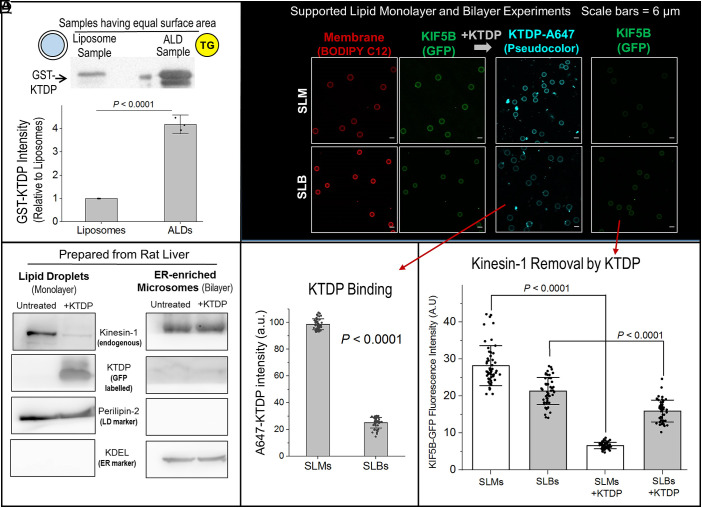
KTDP preferentially binds to and removes kinesin-1 from monolayer membranes in-vitro. (*A*) ALDs and Liposome samples are normalized to have approximately equal surface area (see main text). Samples are incubated with GST-KTDP, then separated from unbound GST-KTDP by centrifugation. Immunoblotting with GST antibody shows ~fourfold higher binding of GST-KTDP to ALDs. Data represent mean ± SD of three independent experiments. (*B*) LD (monolayer) and ER-enriched microsome (bilayer) samples are prepared from the rat liver. Each sample is divided into two equal parts. One part is left untreated and the other treated with GFP-KTDP. LDs or microsomes are separated from unbound GFP-KTDP by centrifugation. Immunoblotting against GFP shows significant binding of KTDP to LDs, but no detectable binding to microsomes. Perilipin-2 and KDEL immunoblots respectively show purity of LD and microsome samples. These immunoblots also show equal loading of LDs and microsomes across untreated and KTDP-treated lanes. (*C*) Hydrophobic or hydrophilic beads are incubated with BODIPY labeled liposomes (made from 95% PC + 5% PA). This results in formation of SLMs or SLBs with a fluorescent (BODIPY) membrane around them. SLMs were made using 6.4 µm (dia) DVB coated latex beads. SLBs were made using 5.7 µm (dia) carboxylated latex beads. SLMs and SLBs are then incubated with GFP tagged Kinesin-1 (GFP-KIF5B) to detect kinesin-1 recruitment on them in Confocal images. SLMs and SLBs are then treated with A647-labeled KTDP. KTDP-treated SLMs and SLBs are imaged to detect A647-KTDP and GFP-KIF5B on these structures. (Scale bar, 6 μm.) (*D*) A647-KTDP fluorescence intensity on SLMs is significantly higher than SLBs, showing higher affinity of KTDP to SLMs. Each data point represents the integrated intensity of A647 fluorescence in a circular periphery around a single SLB or SLM. Error bars are Mean ± SD. (*E*) GFP-KIF5B fluorescence intensity on SLMs and SLBs imaged before and after treatment with A647-labeled KTDP. Each data point represents the integrated intensity of GFP fluorescence in a circular periphery around a single SLB or SLM. Error bars are Mean ± SD.

We first tested for the binding affinity of KTDP in a minimal reconstituted system using liposomes (these have a bilayer membrane) and artificial LDs (ALDs) that have a monolayer phospholipid membrane around a TG core (7). Because KTD binds to PA, liposomes and ALDs were both prepared using phosphatidylcholine (PC, 95%) and PA (5%). The ALD and liposome samples were normalized using the amount of PC detected on them by a thin layer chromatograph (*SI Appendix*, Fig. S1*E*). For the same total surface area, a monolayer-LD should have approximately half of the PC content of a bilayer-liposome. Thus, we used appropriate dilutions to prepare an ALD sample with half the amount of PC as compared to a liposome sample, expecting these samples to have approximately same membrane surface available for KTDP binding (*SI Appendix*, Fig. S1*E*). These samples were incubated with a GST-tagged KTDP prepared in bacteria (GST-KTDP; *SI Appendix*, Fig. S1*D*), followed by centrifugation to separate ALDs and liposomes. Western blotting against GST demonstrated fourfold higher intensity of GST-KTDP on ALDs as compared to liposomes (Fig. 2*A*). We will verify this preference of KTDP to monolayer membranes at single-vesicle level by optical microscopy in the next section.

### KTD Peptide Displaces Endogenous Kinesin-1 More Effectively from Monolayer Membranes.

If KTDP binds preferentially to monolayers, then can it also displace kinesin-1 from LDs with higher efficiency as compared to bilayer organelles? To test this, we purified LDs and ER-enriched microsomes (i.e., bilayer vesicles) from the rat liver. The LD sample and the microsome sample were each divided into two equal parts. One part was left untreated and the other was treated with GFP-tagged KTDP prepared in bacteria. LDs or microsomes were then separated from unbound proteins by centrifugation, and analyzed by western blotting. GFP-KTDP was recruited abundantly to LDs, concomitant with significant loss of kinesin-1 from LDs (Fig. 2*B*). Contrastingly, GFP-KTDP was barely detectable on microsomes, which retained almost all of their kinesin-1 even after KTDP treatment (Fig. 2*B*). Equal loading of untreated and KTDP-treated LDs in western blot samples was verified by TG content (*SI Appendix*, Fig. S1*F*). Fig. 2*B* also shows western blots against perilipin-2 (LD marker) and KDEL (ER marker) which confirm the purity of LD and microsome samples. The similar intensities of perilipin-2 and KDEL shows equal loading of LDs and microsomes in untreated and KTDP-treated western blot samples (Fig. 2*B*).

The above-described LDs/ALDs/microsomes are appropriate for bulk biochemical experiments, but their small size (<1 µm) precludes their use for optical imaging. To verify by direct imaging the effect of KTDP at a single-vesicle level, we developed an in-vitro assay as described in *SI Appendix*, Fig. S1*G*. We used large latex beads that were either hydrophobic (DVB-coated; 6.4 µm diameter) or hydrophilic (Carboxylated; 5.7 µm diameter). Liposomes with lipid composition (95%PC + 5%PA) were prepared and doped with a trace amount of fluorescent BODIPY-C12. These liposomes were then deposited onto the hydrophobic or hydrophilic beads to respectively create supported lipid monolayers (SLMs) or supported lipid bilayers (SLBs). BODIPY-C12 fluorescence imaging confirmed tight circular membranes on SLMs and SLBs (Fig. 2*C*). Fluorescence measured along a circular profile expectedly showed lower average intensity for the monolayer on SLMs than the bilayer on SLBs (*SI Appendix*, Fig. S1*H*). SLMs and SLBs were then adjusted by dilution to obtain samples that had the same optical density, and therefore the same number of beads/unit volume. We verified this normalization by visual counting of SLMs and SLBs using a hemocytometer. Because these two kinds of beads have approximately same diameter and they are present in equal number in the normalized samples, the total membrane surface area available for KTDP binding should be similar for SLM and SLB samples.

Cytosol was next prepared from cells overexpressing GFP-tagged kinesin-1 (GFP-KIF5B). SLMs and SLBs were incubated separately with cytosol to recruit GFP-KIF5B, then separated from cytosol via centrifugation. Confocal images showed that GFP-KIF5B was recruited to both SLMs and SLBs (Column 2, Fig. 2*C*). These SLMs and SLBs were then treated with Alexa-647 labeled GST-KTDP, separated by centrifugation and imaged again. Alexa-647 signal on SLMs was significantly higher than SLBs (Column 3, Fig. 2*C*). Quantification showed ~fourfold higher Alexa-647 fluorescence per unit surface area on SLMs compared to SLBs (Fig. 2*D*). This finding is in excellent agreement with the ~fourfold higher KTDP detected on ALDs by western blotting (as compared to liposomes; Fig. 2*A*). Thus, KTDP has ~fourfold higher affinity for the monolayer membrane, and might therefore be able to displace kinesin-1 more effectively from monolayers. To test this, GFP-KIF5B fluorescence was measured along the periphery of individual SLMs and SLBs (Fig. 2*E*; at least 50 per condition). The below described ratios (*R*_SLM_, *R*_SLB_) were defined and measured from the images:-

$$R_{\text{SLM}}=\frac{\mathrm{Average}\;\mathrm{GFP}\;\mathrm{Fluorescenceon}\;\mathrm{SLMs}\;\mathrm{After}\;\mathrm{KTDP}\;\mathrm{treatment}}{\mathrm{Average}\;\mathrm{GFP}\;\mathrm{Fluorescenceon}\;\mathrm{SLMs}\;\mathrm{Before}\;\mathrm{KTDP}\;\mathrm{treatment}}=0.23\pm 0.05,$$

$$R_{\text{SLB}}=\frac{\mathrm{Average}\;\mathrm{GFP}\;\mathrm{Fluorescenceon}\;\mathrm{SLBs}\;\mathrm{After}\;\mathrm{KTDP}\;\mathrm{treatment}}{\mathrm{Average}\;\mathrm{GFP}\;\mathrm{Fluorescenceon}\;\mathrm{SLBs}\;\mathrm{Before}\;\mathrm{KTDP}\;\mathrm{treatment}}=0.72\pm 0.19.$$

Thus, only ~23% kinesin-1 was retained on SLMs after KTDP treatment (77% was removed). In contrast, ~72% kinesin-1 was retained on SLBs (only 28% was removed). We used these ratios because the GFP signal on SLBs before KTDP treatment was slightly lower than SLMs (Fig. 2 *C* and *E*).

### KTD Peptide Reduces Lipid Secretion from McA-RH7777 Cells.

**Fig. 3.**
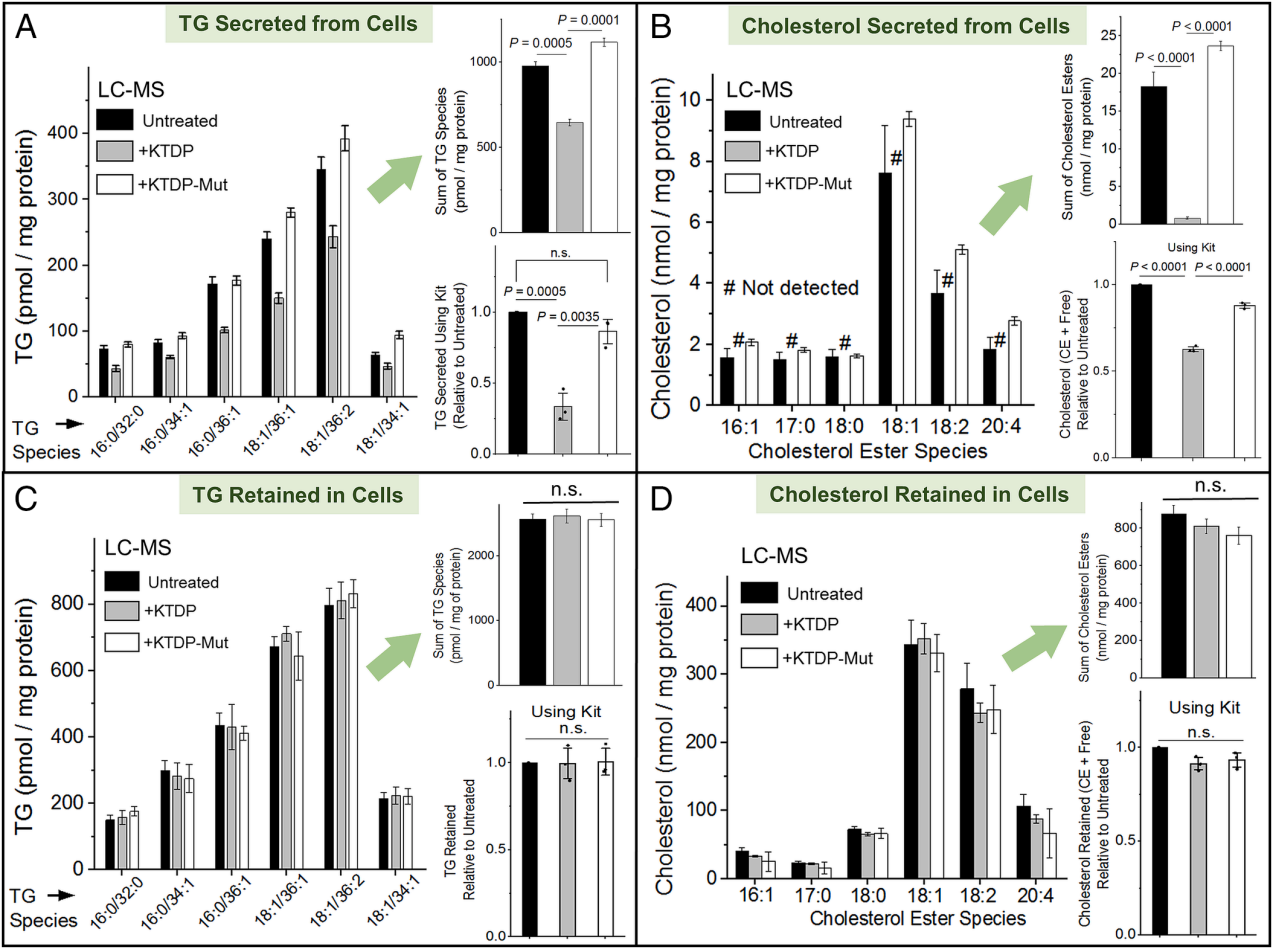
Effect of KTDP on secretion and retention of lipids (McARH-7777 hepatoma cells). (*A*) LC-MS for TG in secreted media of untreated, GFP-KTDP, and GFP-KTDP-mut overexpressing cells. The major species of detected TGs are shown. *Upper* *Inset* shows the sum of detected TG species in LC-MS. Errors have been propagated. An overall reduction of ~50% secreted TG is apparent from KTDP treated cells. *Lower* *Inset* Similar reduction is also observed using a commercial TG assay kit. Error bars are SEM. (*B*) LC-MS for Cholesterol in secreted media of untreated, GFP-KTDP, and GFP-KTDP-mut overexpressing cells. The major species of detected cholesterol esters (expected to be present inside lipoprotein particles) are shown. Cholesterol ester secreted from KTDP treated cells was below the background levels in LC-MS data. *Upper* *Inset* shows the sum of detected cholesterol ester species in LC-MS. Errors have been propagated. A drastic reduction of secreted cholesterol esters is apparent from KTDP treated cells. *Lower* *Inset* Secreted cholesterol reduction is also observed using a commercial cholesterol assay kit that measures Esterified + Free cholesterol. Error bars are SEM. (*C*) LC-MS for TG retained inside untreated, GFP-KTDP, and GFP-KTDP-mut overexpressing cells. The major species of detected TGs are shown. *Upper* *Inset* shows the sum of detected TG species in LC-MS. Errors have been propagated. No significant difference in TG retention is apparent across conditions. *Lower* *Inset* A TG assay kit also detects no significant difference across conditions. Error bars are SEM. (*D*) LC-MS for Cholesterol retained inside untreated, GFP-KTDP, and GFP-KTDP-mut overexpressing cells. The major species of detected cholesterol esters are shown. *Upper* *Inset* shows the sum of detected cholesterol ester species in LC-MS. Errors have been propagated. No difference in cholesterol retained inside cells is observed across conditions. *Lower* *Inset* *A* commercial cholesterol assay kit that measures Esterified + Free cholesterol also detects no difference across conditions. Error bars are SEM.

If KTDP removes kinesin-1 efficiently from LDs, then does it also reduce lipid secretion from cells? To check this, we measured TG and CE secreted into medium from McA-RH7777 hepatoma cells after overexpressing KTDP or a KTDP mutant that is defective for PA-binding (7, 30). These cells are well known to secrete VLDL (7, 22, 29). Both peptides were expressed at equal levels in these cells (*SI Appendix*, Fig. S1*C*), as also seen earlier (7). Cells were first pulsed with oleic acid (OA) to provide a substrate for LD formation. The OA-containing medium was removed, then cells were chased in serum-free medium. The medium was collected by aspiration after incubation, and the cells were washed and harvested separately. LC-MS lipidomics of the collected medium showed that KTDP significantly reduced the secretion of specific long-chain TG species as compared to untreated and KTDP-mutant overexpressing cells (Fig. 3*A* and *Upper* *Inset* Fig. 3*A*). Measurement using a commercial TG assay kit confirmed ~50% reduction of TGs in the medium (*Lower* *Inset*, Fig. 3*A*). LC-MS also confirmed a drastic reduction of cholesterol esters in secreted medium (Fig. 3*B* and *Upper* *Inset*, Fig. 3*B*). Measurement with a commercial cholesterol assay kit (detects both ester and free cholesterol) confirmed significant reduction of secreted cholesterol in medium upon KTDP overexpression (*Lower Inset*, Fig. 3*B*).

KTDP expression caused no increase in alanine aminotransferase (ALT) activity in McA-RH7777 cells, suggesting KTDP does not cause any toxicity (*SI Appendix*, Fig. S2*A*1). We could also not detect any obvious change in cell morphology after KTDP expression (Fig. 1*D* and *SI Appendix*, Fig. S2*A*2). We have observed no change in cell proliferation by BrdU staining across untreated, KTDP and KTDP-mutant treated conditions earlier (7). Similar level of TG was detected inside untreated and KTDP-treated cells after a 12-h pulse of Oleic acid, suggesting no significant effect of KTDP on LD biogenesis (*SI Appendix*, Fig. S2*B*1). Further, no change in cellular phospholipids such as PC and Lyso-PC was caused by KTDP treatment (*SI Appendix*, Fig. S2*B*2).

### KTD Peptide Does Not Cause Lipid Accumulation in McA-RH7777 Cells.

Very interestingly and surprisingly, LC-MS measurements and a commercial assay kit both showed that KTDP caused no increase of TG (Fig. 3*C* and *Insets* of Fig. 3*C*) or CE (Fig. 3*D* and *Insets* of Fig. 3*D*) inside McA-RH7777 cells. To understand this result, we investigated the metabolic fate of cellular lipids under these conditions. Intriguingly, the oxygen consumption rate (OCR) of KTDP treated cells was higher (*SI Appendix*, Fig. S2*C*), suggesting increased β-oxidation in mitochondria. LC-MS measurements revealed that KTDP also increases free fatty acids (FFAs) retained inside cells marginally (*SI Appendix*, Fig. S2*D*), but causes no change in FFAs secreted from cells (*SI Appendix*, Fig. S2*E*). These results may suggest that KTDP induces a lipolytic pathway, causing TG to be broken down into FFAs, which accumulate transiently inside cells before they are trafficked to mitochondria for β-oxidation. To test this possibility, we quantified the lipid flux between LDs and mitochondria using a pulse–chase approach (33). Untreated or KTDP-expressing cells were pulsed with a trace amount of Bodipy-C12 (12-Carbon fatty acid conjugated to BODIPY 558/568 fluorophore). Cells were then chased for 16 h followed by imaging of the live cells. In untreated (control) cells, Bodipy-C12 fluorescence was localized to LDs after the chase, indicating retention of labeled fatty acids in neutral lipid pools (*SI Appendix*, Fig. S3*A*). In contrast, KTDP-overexpressing cells exhibited marked reduction in LD associated Bodipy-C12, together with substantial enrichment of fluorescence within mitochondria (*SI Appendix*, Fig. S3*A*). Colocalization analysis demonstrated strong overlap of the Bodipy-C12 with mitotracker-positive mitochondrial structures (*SI Appendix*, Fig. S3*B*) upon KTDP overexpression, consistent with enhanced lipid trafficking from LDs to mitochondria in this condition.

If indeed KTDP-expressing cells prevent LD accumulation by rerouting lipids from LDs to mitochondria, then blocking lipid uptake into mitochondria should cause lipid accumulation even in the presence of KTDP. To test this, untreated (control) or KTDP-expressing cells were treated with etomoxir, an inhibitor of carnitine palmitoyltransferase-1 (CPT-I), which blocks mitochondrial fatty acid import. Etomoxir increased LD number in control cells by ~1.5-fold, but by ~2.5-fold in KTDP-overexpressing cells (*SI Appendix*, Fig. S3 *C* and *D*). KTDP-expressing etomoxir-treated cells also had LDs of the largest size (*SI Appendix*, Fig. S3*C*). All these findings indicate that the absence of lipid accumulation under KTDP overexpression likely stems from rerouting of LD-derived fatty acids towards mitochondrial fatty acid oxidation, as also reflected in increased mitochondrial oxidative activity (*SI Appendix*, Fig. S2*C*). The simplest explanation for such rerouting is that KTDP inactivates LD transport to redistribute LDs from the hepatocyte periphery to all over the cell, thus bringing LDs into close proximity with mitochondria that are similarly distributed across the cell (see *Discussion* for further thoughts on this aspect). If this rerouting holds true in-vivo, then it has the implication that circulating serum-TG and Cholesterol can be lowered by KTDP without the undesirable side-effect of fatty liver in an animal. We will explore this exciting possibility in the next sections.

### KTD Peptide Can Be Delivered to the Liver of Zebrafish Larvae Using Egg Liposomes.

We next tested the effect of KTDP in a whole-organism model. We chose zebrafish (*Danio rerio*), an established model for studying hyperlipidemia and metabolic disorders (27, 28). The almost-transparent larvae permit in-vivo imaging of lipid distribution across visceral organs (e.g., liver, intestine) and the vasculature (26). Further, only a few species (Zebrafish, Humans, Rabbits, Hamsters) have an ortholog of the Cholesteryl Ester Transfer Protein (CETP), a gene that is not found in rodents. Because CETP transfers neutral lipids between plasma lipoprotein particles (VLDL, HDL, and LDL), the circulating lipoproteins in zebrafish closely resemble their human counterparts in composition and abundance. Zebrafish may therefore be more suitable than rodents to study the ill-effects of lipoprotein dysregulation. Indeed, atherosclerotic lesions are formed in zebrafish kept on a high-cholesterol diet (34), but harder to observe in rodents.

Our objective was to test whether KTDP can reduce circulating lipids in zebrafish larvae. For the purpose of peptide delivery, we prepared liposomes using the yolk of chicken egg. Egg-liposomes have been used to deliver lipids and monitor lipid fluxes in zebrafish; they are also a simple method for simulating high-fat diet conditions in zebrafish (35). However, to best of our knowledge, egg-liposomes have never been used for peptide delivery to zebrafish. Peptides corresponding to human KTDP were used in zebrafish as they are highly conserved (*SI Appendix*, Fig. S1*A*). The PA-binding region of zebrafish KTD is identical to humans except for a single S→A change (Fig. 1*B*).

Three kinds of liposomes were prepared: No peptide (Empty), loaded with GFP-KTDP, or loaded with a GFP-KTDP mutant that cannot bind PA. The latter two conditions used 250 µg/mL concentration of peptide in the solution when preparing liposomes. Strong GFP signal appeared within nearly all liposomes of the last two categories, indicating successful peptide incorporation inside liposomes (*SI Appendix*, Fig. S4*A*). These liposomes were labeled with trace amounts of BODIPY-C12, a fluorophore conjugated to a fatty acid chain that gets incorporated into TG and thus allows lipid visualization in larvae (36). Zebrafish embryos rely on yolk fats for nutrition until 5 days postfertilization (5 dpf), after which they consume external food (27). We therefore used 6 dpf larvae for our studies. Liposomes were mixed into the water in which larvae were swimming (i.e., larval medium), with the expectation that larvae would eat up the nutrient-rich liposomes along with the KTDP inside liposomes. A schematic of the liposomal KTDP delivery experiments is shown in *SI Appendix*, Fig. S4*B*.

**Fig. 4.**
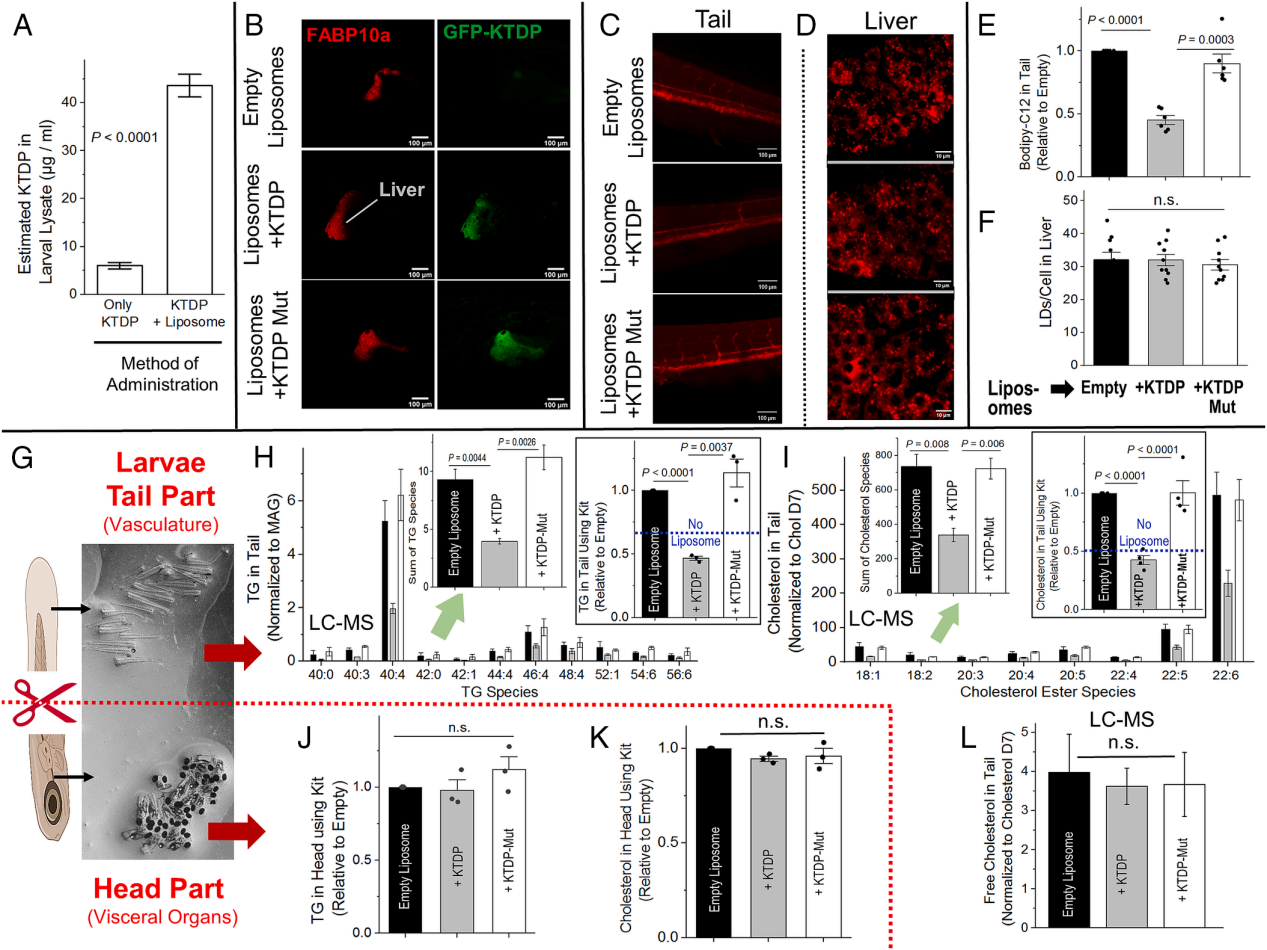
Liposomal delivery of KTDP to zebrafish larvae: Effect on lipid content in tail and head parts. (*A*) Quantification of GFP fluorescence in zebrafish lysates prepared from larvae (6 dpf) fed with GFP-KTD without liposomes (250 µg/mL), or GFP-KTD packaged in Egg liposomes. Calibration curve was used for estimating KTDP amount from GFP fluorescence (see main text). Error bars are Mean ± SEM. *N* = 3. (*B*) Confocal images of zebrafish larvae after liposomal feeding of GFP-KTDP. Liver is marked in red in fish expressing FABP10a-mCherry (Red; liver marker). (Scale bar, 100 μm.) (*C*) Confocal z-stacked images of the tail of larvae fed with BODIPY-C12 labeled liposomes of three types:- Empty liposomes, with KTDP or with KTDP-Mut. (Scale bar, 100 μm.) (*D*) Confocal images of Nile Red staining in the liver of larvae fed with liposomes as above. (Scale bar, 10 μm.) (*E*) Quantification of BODIPY-C12 fluorescence in KTDP-treated larval tail (mean ± SEM, N = 6). (*F*) Counting of Nile Red stained LDs inside hepatocytes in the larval liver shows no effect of KTDP (mean ± SEM, N = 10). (*G*) Larvae fed with liposomes (Empty, +KTDP or +KTDP-Mut) are bisected. The head (contains visceral organs e.g., liver, intestine, etc.) and tail regions (vasculature) are separated and pooled for lipid estimation. We pooled 30 larval heads and 30 tails to make up a single replicate. Three such replicates were made for each condition of liposome treatment. Thus, a total of 30 × 3 × 3 (=270) larval heads and 270 larval tails were used across the different replicates and treatments. (*H*) LC-MS for secreted TG in pooled larval tails. *Inset*:- shows the sum of detected TG species. Errors have been propagated. An overall reduction of >50% secreted TG is apparent in KTDP-fed larvae. Boxed *Inset*:- Similar reduction is observed using a TG assay kit. The data are normalized to TG values for larvae fed with empty liposomes. The blue dashed line represents TG measured for untreated larvae (no liposomes; no KTDP). Error bars are SEM. (*I*) LC-MS for secreted cholesterol in pooled larval tails. *Inset*:- Sum of all detected cholesterol ester species. Errors have been propagated. An overall reduction of >50% secreted cholesterol is apparent in KTDP-fed larvae. Boxed *Inset*:- Similar reduction is observed in the tail using a cholesterol assay kit. The data are normalized to cholesterol values for larvae fed with empty liposomes. The blue dashed line represents cholesterol measured for untreated larvae (no liposomes; no KTDP). Error bars are SEM. (*J*) Measurement using a TG assay kit shows no effect of KTDP on TG content in the head part of larvae (contains visceral organs). Error bars are SEM. (*K*) Measurement using Cholesterol assay kit suggests that KTDP causes no change in Cholesterol in the head part of larvae. Error bars are SEM. (*L*) LC-MS shows no effect of KTDP on Free cholesterol in tail part (vasculature) of larvae. Error bars are SEM.

Liposomes were administered in aforesaid manner over a 6-h period with gentle agitation. Similar BODIPY-C12 signal was seen across different conditions immediately after this duration at the whole-larva level and inside the gut (*SI Appendix*, Fig. S4*C*). We therefore believe that larvae consumed the liposomes in similar quantity across conditions. We next asked whether the liposomes are actually helping in KTDP delivery to the larvae. GFP fluorescence was measured on serially diluted GFP-KTDP to prepare a calibration curve (*SI Appendix*, Fig. S4*D*). We then measured GFP fluorescence in larval lysate prepared from larvae that had been fed with GFP-KTDP packaged inside liposomes, or GFP-KTDP simply mixed into water without any liposomes (again at 250 µg/mL concentration). Fig. 4*A* shows the estimated KTDP concentration in whole larval lysate using the calibration curve of *SI Appendix*, Fig. S4*D*. Barely any fluorescence was seen for KTDP without liposomes. Thus, the egg-liposomes do act as an effective peptide delivery agent to larvae. We next used transgenic zebrafish expressing the liver-specific marker FABP10a-mCherry to visualize the larval liver (Fig. 4*B*). Liposomal delivery of GFP-KTDP or GFP-KTDP mutant caused robust GFP signal colocalizing with FABP. It therefore appears likely that some of the administered peptide is delivered to the larval liver without significant degradation because GFP fluorescence should have been lost had the peptide been degraded and/or if its structure had been significantly altered.

### KTD Peptide Reduces Circulating Lipids in Zebrafish Larvae.

Because the larval tail is devoid of visceral organs (28), BODIPY-C12 imaging in the tail allowed us to visualize lipids that had been secreted out into vasculature. KTDP reduced BODIPY-C12 fluorescence by ~50% in the tail as compared to empty liposome or KTDP-mutant treated larvae (Fig. 4 *C* and *E*). Confocal imaging by Nile Red staining in the larval liver showed no accumulation or abnormal morphology of LDs or change in LD number after KTDP feeding (Fig. 4 *D* and *F*). We therefore believe that KTDP had no significant effect on LD biogenesis in the zebrafish liver. These findings are reminiscent of the unchanged TG and CE inside McA-RH7777 cells after KTDP expression (Fig. 3 *C* and *D* and *SI Appendix*, Fig. S2*B*1).

We next measured the TG in the larval head part (contains visceral organs) and tail (contains vasculature) using biochemical methods. To do this, zebrafish larvae were fed with liposomes for 5 h. Larvae were then washed, and individual larvae bisected to separate out the head and the tail parts (Fig. 4*G*). We pooled 30 larval heads and 30 tails to make up a single replicate, and then measured TG and Cholesterol in each replicate. TG in KTDP treated larval tail was reduced by ~50%, as seen by LC-MS (Fig. 4*H*) and also using a TG assay kit (Boxed *Inset*, Fig. 4*H*). On similar lines, KTDP caused ~50% reduction of cholesterol esters in the larval tail (Fig. 4*I*), but no change in free cholesterol in the tail (Fig. 4*L*). These findings are in excellent agreement with the reduction of neutral lipids estimated by BODIPY fluorescence in the larval tail (Fig. 4 *C* and *E*). Again, reminiscent of the observation in McA-RH7777 cells, KTDP caused no increase of TG (Fig. 4*J*) or cholesterol (Fig. 4*K*) in the head part of the larvae.

### Time Course of Lipid Reduction, Phenotype, Mortality, and Locomotion of Larvae after KTDP Feeding.

How long does the lipid-lowering effect of KTDP last, and are there unwanted side-effects beyond this period? To explore, we fed 6 dpf larvae with liposomes that were Empty, filled with KTDP or with KTDP-Mutant. Larvae were fixed at 6, 12, 24, 48, and 120 h post liposome administration, then stained with Nile Red for imaging neutral lipids. We did not use BODIPY-C12 labeled liposomes because this dye gets metabolized and is not visible after 12 h. *SI Appendix*, Fig. S5*A* shows the staining and *SI Appendix*, Fig. S5*B* quantifies change in Nile Red fluorescence at different time points. A significant reduction in lipids was seen in the tail (vasculature) of KTDP-fed larvae at 6 and 12 h compared to empty or KTDP-mutant fed larvae, followed by return to baseline levels (*SI Appendix*, Fig. S5*B*).

KTDP-fed larvae showed no observable deformity or abnormality up to 48 h postfeeding as compared to empty or KTDP-Mut fed larvae (*SI Appendix*, Fig. S6*A*). Mortality of larvae was similar across experimental groups up to 120 h post KTDP feeding (*SI Appendix*, Fig. S6*B*). To investigate potential long-term effects on larval locomotion, we stimulated larvae at the center of a petri dish with a micropipette tip 120 h after KTDP feeding. We then measured where larvae first stopped after stimulation across concentric zones (*SI Appendix*, Fig. S6*C*). No significant difference in first-stop zone was observed across conditions (*SI Appendix*, Fig. S6*D*). Free-swimming larvae were also imaged 120 h after KTDP feeding, and their motion tracked over 6 min (*SI Appendix*, Fig. S6*E*). Analysis of tracks showed no differences in total distance traveled (*SI Appendix*, Fig. S6*F*), or angular velocity during motion (*SI Appendix*, Fig. S6*G*) across conditions. To sum up, we could observe no obvious effect of KTDP on the phenotype, mortality, and locomotion of zebrafish larvae.

### Oral Delivery of KTDP to Adult Zebrafish: Effects on Serum and Liver Lipids.

Because oral consumption is a preferred method for drug delivery, we attempted to deliver egg-liposomes containing KTDP to adult (1 y old) Zebrafish by oral gavaging. A gavaging procedure to deliver infectious agents to zebrafish (37) was adapted to deliver KTDP packaged inside egg liposomes (5 µL of liposomes per fish; see *Materials and Methods*). Egg liposomes labeled with BODIPY-C12 (Empty controls, or loaded with KTDP) were administered by gavaging for three consecutive days at a KTDP concentration of 250 µg/mL, identical to the dose used in larval studies. Fish displaying bleeding, distress, or abnormal behavior were excluded from further experimentation. Fish were euthanized 4 h after the final administration according to institutional guidelines. The tail (vasculature) was dissected out and centrifuged to collect blood and serum. The liver and anterior gut tissues were also dissected out for imaging and biochemical studies.

**Fig. 5.**
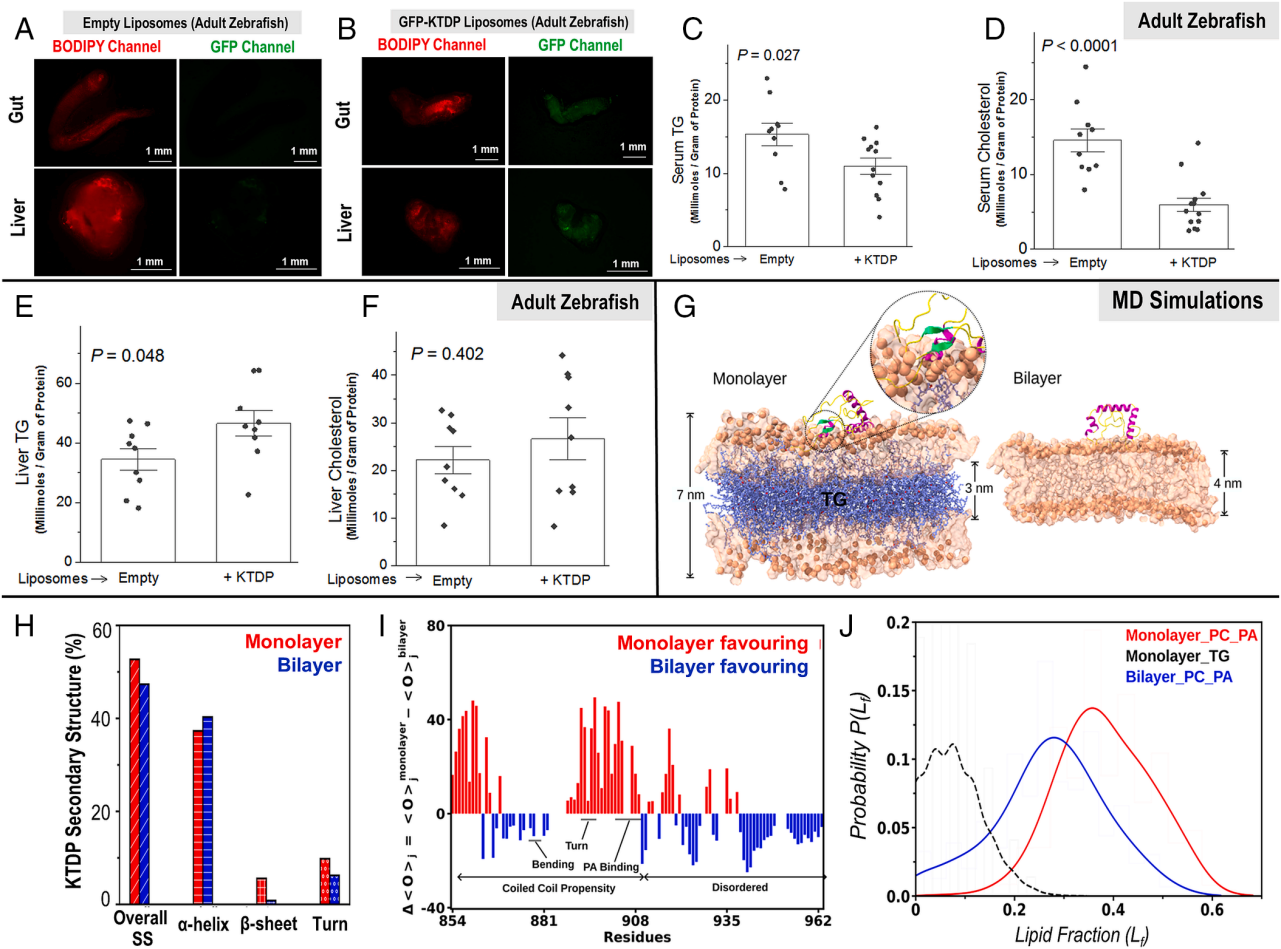
Lipid-lowering effect of KTDP in adult zebrafish and MD simulation of KTDP–membrane interactions. (*A*) Confocal image of the Gut (intestine) and Liver dissected out from 1-y-old zebrafish. The fish were fed for 3 d with Empty egg-liposomes (containing no peptide) by an Oral gavaging method. Liposomes were labeled with trace amounts of BODIPY-C12 for visualization. (*B*) Same as *A*, but with liposomes containing GFP-KTDP. GFP signal colocalizing with BODIPY suggests that the peptide was delivered intact to gut and liver tissues of fish. (*C*) TG in blood (serum) obtained from the tail part (vasculature) of adult fish that were treated with Empty or KTDP-containing liposomes. Measurement was done using a commercial TG assay kit. Each data point represents a single fish. Error bars are SEM. (*D*) Cholesterol TG in blood obtained from the tail part (vasculature) of adult fish that were treated with Empty or KTDP-containing liposomes. Measurement was done using a commercial TG assay kit. Each data point represents a single fish. Error bars are SEM. (*E*) TG in dissected liver of adult fish treated with Empty or KTDP-containing liposomes. Measurement was done using a commercial TG assay kit. Each data point represents a single fish. Error bars are SEM. (*F*) Cholesterol in dissected liver of adult fish treated with Empty or KTDP-containing liposomes. Measurement was done using a commercial cholesterol assay kit. Each data point represents a single fish. Error bars are SEM. (*G*) Representations of KTDP-monolayer and KTDP–bilayer conformations in MD simulations. The *Upper* and *lower* leaflets (translucent, orange), phosphorus atoms (orange beads), TG (purple), and KTDP secondary structures (magenta helix, yellow turns, green β-sheets) are shown. The average membrane thicknesses are reported in nanometers. (*H*) Average secondary structure contents of KTDP in monolayer and bilayer systems. (*I*) Difference in the lipid occupancy ($\Delta <O{>}_{j}$) of KTDP residues between monolayer and bilayer systems (see main text). The values on Y axis denote the time fraction of the entire simulation (expressed as a percentage) that a given residue spends in the vicinity of phospholipids (DOPC or DOPA). The calculation did not distinguish between headgroups vs. tails of DOPC or DOPA. Specific regions of KTDP relevant to membrane binding are mentioned. (*J*) Probability distribution of the Lipid fraction (*L_f_*) around monolayer and bilayer favoring residues of KTDP (see main text).

Fig. 5*A* shows the gut and liver tissues that have been dissected out from adult fish gavaged with empty liposomes. BODIPY signal in these tissues confirms that egg liposomes are delivered to the intestine and liver via oral route. Fig. 5*B* shows the corresponding tissues for GFP-KTDP containing liposomes, where the GFP fluorescence suggests that KTDP is delivered to these tissues. Fig. 5 *C* and *D* show a KTDP-induced reduction of TG and Cholesterol in serum of adult fish as compared to Empty Liposomes. Each data point in these figures corresponds to a single adult fish. Fig. 5 *E* and *F* confirm that KTDP causes no abnormal accumulation of TG or cholesterol in the liver of adult zebrafish, in agreement with similar observations in cell culture (Fig. 3) and larvae (Fig. 4). We found no significant difference of AST/ALT ratio in the serum of adult zebrafish across untreated (control), KTDP and KTDP-mutant fed animals, suggesting that KTDP caused no hepatotoxicity in zebrafish (*SI Appendix*, Fig. S6*H*)

### Structural Changes in KTD Upon Membrane Binding.

As shown above, KTDP can remove endogenous kinesin-1 from LDs to reduce TG/Cholesterol secretion from cells. We therefore explored the structural aspects of KTD–membrane interactions. Although crystal structures are not available, earlier analysis suggests significant coiled coil structures that terminate between AA 900-910 of KTD (38). Modeling in I-TASSER (*Materials and Methods*) predicted that KTD contains two N-terminal helices (NH1 and NH2) and a C-terminal intrinsically disordered region (C-IDR), as shown in Fig. 1 *B* and *C*. The helix region (aa 854-913) of KTD is highly conserved and therefore likely to be functionally relevant. A helical wheel representation of this region using Heliquest (39) showed accumulation of hydrophobic and hydrophilic amino acids on opposite sides (*SI Appendix*, Fig. S1*A*, *Right*). It is therefore possible that a conserved function of KTD is to recruit kinesin-1 to LDs by forming an amphipathic helix on the membrane (3, 11). Notably, the NH2 domain of KTD also contains a sequence (AA 901-909) that is rich in positively charged residues and implicated for binding to negatively charged PA on membranes (Fig. 1*B* and *SI Appendix*, Fig. S1*A*).

With this information, we undertook molecular dynamics (MD) simulations of KTDP binding to PA-containing monolayer and bilayer membranes (*Materials and Methods*). Following earlier work (40), we created a monolayer model containing a 3 nm thick slab of TG between two leaflets of phospholipids that were composed of 1,2-dioleoyl-sn-glycero-3-phosphocholine (DOPC) and 1,2-dioleoyl-sn-glycero-3-phosphate (DOPA; phosphatidic acid) taken in a 95:5 ratio (Fig. 5*G*). The bilayer model was made with the same phospholipid composition (Fig. 5*G*). A top view of membrane leaflets of monolayer and bilayer membranes is shown in *SI Appendix*, Fig. S7*A*. As seen earlier (40), the monolayer showed surface-exposed TG molecules that likely cause packing defects, as also reflected in the higher area per lipid (APL) for monolayers (*SI Appendix*, Fig. S7*B*). KTDP was placed near the *Upper* leaflet and MD trajectories of 1.25 μs generated to interrogate the interaction of KTDP with monolayer and bilayer systems. The dynamics of KTDP interacting with monolayer and bilayer systems is shown in Movies S1 and S2.

On average, KTDP acquired higher secondary structure (SS) content in presence of the monolayer (Fig. 5*H*). Structural flexibility in the monolayer was corroborated by enhanced distortion in the helical region (*SI Appendix*, Fig. S7*C*). The loss in helical content on monolayer was offset by the emergence of β-sheets and turn (Fig. 5*H*). Backbone rmsd of KTDP was increased in the bilayer, indicating greater fluctuations and a transient nature of KTDP–bilayer interactions compared to monolayer (*SI Appendix*, Fig. S7*D*). We next calculated the lipid occupancy (*O_ij_*), i.e., the time fraction for which the *j*th amino acid residue of KTDP contacts a lipid indexed by *i* (*i* = DOPC or DOPA). Fig. 5*I* shows the difference in lipid occupancy between monolayer and bilayer systems as a function of *j*:-

$$\Delta <O{>}_{j}\;=\;<O{>}_{j}^{\mathrm{Monolayer}}-<O{>}_{j}^{\mathrm{Bilayer}}.$$

Here ${<O>}_{j}$ is the averaged occupancy of *j*th residue of KTDP across DOPC-DOPA (*Materials and Methods*). Only residues having occupancy >5% are reported. Values of $\Delta <O{>}_{j}$ that are positive (negative) signify higher affinity to monolayer (bilayer) membranes of the *j*th residue. The average value of $\Delta {<O>}_{j}$ for monolayer favoring residues is ca. 23% and bilayer favoring residues is ca. 11%. Thus, overall KTDP–membrane interactions are higher for the monolayer system. We define the Lipid fraction as $L_{f}=\frac{1}{N}\times L_{n}$ where *N* is number of KTDP residues interacting with $L_{n}$ lipids (DOPC or DOPA or TG) within a cutoff distance of 0.7 nm. The probability distribution ${P(L}_{f})$ shows that residues favoring the monolayer interact more with lipids than residues favoring bilayer (Fig. 5*J*). Considering only the nonzero lipid occupancy values, ca. 68% of the TG-interacting KTDP residues are also highly occupied with DOPC/DOPA (*SI Appendix*, Fig. S7*E*), underlining the influence of TG in orchestrating lipid–KTDP interactions in the monolayer. In summary, the presence of interdigitated TG molecules enhances the APL of the monolayer membrane to create surface defects. The less compact membrane sustains interactions, causing SS gain and binding of KTDP to the monolayer.

## Discussion

Elevated plasma lipids are associated with diabetes, cardiovascular disease, and the metabolic syndrome. Reducing atherogenic plasma lipoproteins is the most accepted strategy against these ailments. Fibrates, omega-3 poly-unsaturated fatty acids (PUFAs) and Niacin are prescribed against hyperlipidemia, but their ill-effects are well known (14, 15). Fibrates activate peroxisome proliferator-activated receptor-α (PPARα), a transcription factor regulating numerous genes in mitochondrial fatty acid oxidation. PUFAs down-regulate the SREBP-1c transcription factor and activate PPARα, they also stimulate LPL-mediated lipolysis and apoB degradation to increase VLDL clearance. Niacin inhibits adipose lipolysis, inhibits DGAT2 and reduces expression of apolipoprotein C-III for faster clearance of TG rich lipoproteins. Niacin however also causes liver damage and gastrointestinal problems. Statins effectively reduce cholesterol (and TG to some extent), but subjects still have a significant risk of cardiovascular disease requiring adjunctive therapy. Lomitapide, an inhibitor of the microsomal triglyceride (TG) transfer protein reduces TG assimilation into VLDL and chylomicrons, but also causes hepatic steatosis. Lipase inhibitors have been known for many decades, but Orlistat is the only approved drug of this class. Orlistat provides only mild improvements in total cholesterol and lipoproteins (13). To sum up, most lipid-lowering therapies act via multiple known/unknown cellular pathways and have limited effect. Combination therapies are therefore often needed, thus amplifying their side-effects (14, 15). This situation underlines the need for alternate management strategies against hyperlipidemia.

The kinesin-dependent delivery of LDs for VLDL assembly has been discovered by us in recent years (7, 21, 22), and has therefore never been explored against hyperlipidemia. The possibility that a peptide can remove an endogenous protein from the LD monolayer with minimal interference on bilayer vesicles is a conceptual advance that could be employed against other LD proteins. Particularly, other CYTOLD proteins that bind to LDs using amphipathic helices could be targeted. Kinesin driven LD transport for TG and cholesterol delivery towards VLDL assembly has been demonstrated in human hepatoma cells, mouse primary hepatocytes, and inside the rat liver (7, 22). Here we showed that KTDP can be delivered to the liver of zebrafish larvae and adults using egg liposomes, causing significant reduction of serum-TG and cholesterol (Figs. 4 and 5). KTDP is part of endogenous kinesin-1, and therefore this peptide is unlikely to cause adverse immune responses even at higher dosage. Unlike the MTTP inhibitor Lomitapide that causes fatty liver, KTDP caused no increase of LDs in McA-RH7777 cells and zebrafish liver. KTDP had no effect on LD biogenesis because TG amount retained inside cells was unchanged (Fig. 3*C* and *SI Appendix*, Fig. S2-B1).

As discussed earlier, kinesin-1 activity localizes LDs in hepatocytes to the cell periphery to promote channeling of LDs for VLDL assembly. These LDs also have high activity of ADP-ribosylation factor 1 (ARF1; removes phospholipids from LDs) and Phospholipase D1 (PLD1; generates PA on LDs). These two enzymes respectively make the LD membrane highly “reactive” and fusogenic (7). Kinesin-1 likely ensures minimal interaction of such reactive LDs with mitochondria by rapidly delivering LDs to the peripheral sER. When KTDP inactivates kinesin-1 on LDs, LDs and mitochondria are both distributed all over the cell, likely allowing frequent interaction of reactive LDs with mitochondria, and consequent LD clearance via mitochondrial lipid utilization. This effect of KTDP is supported by increased oxygen consumption (*SI Appendix*, Fig. S2*C*), increased LD-to-mitochondria lipid trafficking (*SI Appendix*, Fig. S3*A*) and increased LD accumulation after etomoxir treatment of KTDP-expressing cells (*SI Appendix*, Fig. S3*D*). The rerouting of LD contents for mitochondrial uptake by KTDP likely prevents lipid accumulation in hepatocytes. Such an effect of KTDP also appears to operate in the zebrafish model, where no lipid accumulation in the liver is caused by KTDP (Fig. 4 *D*–*F*). It is further reassuring to see no developmental, phenotypic, or locomotory defects in zebrafish for significantly long periods after KTDP feeding (*SI Appendix*, Fig. S6).

Kinesin-1 not bound to cargoes inside cells may exist in a folded state where KTD binds to, and blocks the ATPase activity of kinesin’s motor domain (31). Accordingly, a KTD peptide also blocks ATPase activity of kinesin in-vitro. The overexpressed GFP-KTDP in our experiments is ~1% of the total protein content of cells (*SI Appendix*, Fig. S1*C*). Even at such high concentration, why does KTDP have no effect on bilayer organelles? As demonstrated elegantly by others, KLC acts as an activator that prevents the KTD from inhibiting kinesin (32). KLC-mediated activation of kinesin on bilayer organelles may thus make them immune to KTDP. Because KLC overexpression had no effect on LD distribution (Fig. 1*E*), KLC appears irrelevant to kinesin function on LDs. Further, PA is enhanced dramatically on LDs in the liver in fed state by the activity of phospholipase-D1 (7), causing vigorous kinesin-1 driven LD motion towards the smooth-ER (21). KTDP cannot bind LDs when charged residues in the PA-binding domain of KTDP are mutated (7). Thus, PA is essential for KTD/kinesin-1 binding to LDs. The generation of PA on hepatic LDs via phospholipase-D1 activity may be a signal for KTD binding and kinesin activation on LDs. Such a signal is likely absent on bilayer organelles, which use other mechanisms (e.g., KLC) to recruit and activate kinesin-1, and are therefore barely affected by the overexpression of KTDP.

Mutations within a 10-AA PA binding region (Fig. 1*B*) render KTDP ineffective in blocking LD transport and lipid secretion (Figs. 3 and 4). Because this 10-AA region of KTD is critical for LD binding, shorter peptides around this region may also be effective in reducing lipid secretion. We modified the egg-liposome system for delivering the KTDP (with an additional GFP tag) to the liver of zebrafish. The delivered KTDP was functional, as it reduced lipids very effectively in these animals. KTDP was also possibly delivered to the intestine through ingestion of liposomes. Hence, the overall lipid reduction of ~50% could arise via reduced TG/CE secretion from the liver (in the form of VLDL) as well as the intestine (in the form of Chylomicrons). The effects of KTDP on TG/CE supply for chylomicron assembly, if any, remain to be explored. We note encouragingly that liver-targeted delivery of insulin using engineered liposomes is demonstrated (41). Engineered liposomes may further enhance KTDP delivery, and can be tested in rodents and primates. Small molecules mimicking KTDP may also serve this purpose.

To summarize, we earlier showed that kinesin-1 delivers LDs for assembling VLDL particles in hepatocytes. We hypothesized that kinesin-1 may bind LDs using KTD, but via alternate domains to bilayer vesicles. Thus, it is possible to remove kinesin-1 selectively from LDs by using KTDP as a competitive inhibitor (Fig. 1*A*). Indeed, LD transport was inhibited when KTDP was delivered to hepatoma cells, and zebrafish larvae treated with this peptide showed a remarkable reduction of ~50% plasma TG and Cholesterol. Considering that the egg-liposomal delivery system works for delivering KTDP to the liver of zebrafish, we believe that KTDP and its peptidomimetic small molecules are an exciting avenue for exploration. Our results go beyond KTDP and its effect on lipid secretion. The possibility that endogenous proteins can be removed selectively from LDs to modulate metabolic (or other) processes has never been explored, and may be pursued for potential benefits in other pathways of lipid metabolism/LD biology.

## Materials and Methods

### Detailed Methods Are Provided in Accompanying *SI Appendix*.

All reagents used have been described in *SI Appendix*. Zebrafish (*D. rerio*) care and experimental procedures were conducted in accordance with guidelines approved by the Institutional Animal Ethics Committee of IIT Bombay (Approval no. IITB/2025/BSBE/RM01). Sprague-Dawley rats were bred and maintained by the animal house facility at the Tata Institute of Fundamental Research, Mumbai and animal protocols were approved by the Institutional Animal Ethics Committee. HEK-293 T (ATCC CRL-11268) and McA-RH7777 (ATCC CRL-1601) cell lines were used. HEK-293 T cells were cultured in DMEM supplemented with 10% fetal bovine serum (FBS). McA-RH7777 cells were grown in DMEM containing 20% FBS. All cells were maintained at 37 °C in a humidified incubator with 5% CO_2_.

To investigate the helicity of the N-terminal region of KTDP, a helical wheel projection was generated for amino acids 854 to 914 using HeliQuest web server (https://heliquest.ipmc.cnrs.fr/). The sequence was input in single-letter amino acid format, and an α-helical conformation was assumed. GST tagged fusion proteins were expressed in *Escherichia coli* (BL21DE3) and purified using glutathione-conjugated sepharose beads using the manufacturer’s protocol. Briefly, BL21DE3 competent cells were transformed with pGEX-4T1 plasmid containing KTDP and KTDPmut fusion protein gene. GFP-KTDP-His proteins were expressed in *E. coli* BL21(DE3) using 1 mM IPTG induction at OD600 = 0.6 and incubation at 18 °C, overnight. Cells were harvested by centrifugation followed by lysis using sonication in lysis buffer.

ALDs were prepared by using a freeze-thaw technique as described (7). Liposomes were prepared by using a standard freeze-thaw technique (42). For labeling the liposomes, BODIPY-C12 (8 μM) was added to the liposomes, mixed well and kept undisturbed at RT for 30 min. Endoplasmic reticulum enriched microsomes were isolated using the protocol described in ref. 42. LDs were isolated from rat liver using the previously described protocol (7). Lipids from LDs were extracted by the methanol:chloroform method. Extracted lipids were dried, resuspended in chloroform and then loaded onto silica plates prerinsed with chloroform. Separation was performed using two step solvent system i.e., in solvent I (n-hexane/ diethyl ether/acetic acid, 70:30:1) till half way and air dried, then in solvent II (n-hexene/diethyl ether, 59:1) for complete run. After air drying, TG bands were visualized by spraying the plate with 10% CuSO4 in 8% H3PO4, followed by baking >100 °C for 30 min.

Carboxylated latex beads (hydrophilic) of 5.7 μm diameter and Divinylbenzene (DVB) coated latex beads (hydrophobic) of diameter 6.4 μm were used for SLB and SLM preparation respectively. Carboxylated and DVB coated latex beads were added respectively with 1 μL of 1 M NaCl, 69 μL of autoclaved double-distilled water and 20 μL of the BODIPY-C12 labeled liposomes/ These were taken together and kept for 30 min with intermittent vortexing (1 min vortexing with 5 min rest). The mixture was washed thrice by the addition of 1 mL of milliQ water. SLBs and SLMs were pelleted at 10,000 rpm for 5 min and then finally resuspended in 200 μL of double-distilled water.

To assess hepatocellular toxicity effect of KTDP, McARH-7777 cells transfected with GFP-KTDP and GFP-KTDPmut plasmids were measured for ALT enzyme activity using the ALT Colorimetric Activity Assay Kit (Cayman Chemical Cat# 700260). Cellular respiration was measured using Abcam’s extracellular oxygen consumption assay kit (ab197243). TG and total cholesterol measurement were conducted in secreted media and cell lysate using colorimetric assay kits (Elabscience) following the manufacturer’s protocol. Secreted media and cell pellets were used for quantitative LC-MS analysis to measure TG, cholesterol, and FFA following the protocol previously described in ref. 22. Liposome preparation and feeding procedures were conducted following (35), with slight modifications.

### Statistics.

Unless otherwise stated, error bars denote the SEM. Unless otherwise stated, data were assumed to be normally distributed and Student’s *t* test was used to calculate *P*-values for significance (Two-tailed; 95% confidence; Null hypothesis is that distributions are same). The *P* values have been mentioned at most places within figures. At some places following signs denote results of significance tests:- ***(*P* ≤ 0.001), **(*P* ≤ 0.01) and n.s. (*P* > 0.05).

## Supplementary Material

Appendix 01 (PDF)

Movie S1. Video showing the interaction of Kinesin tail domain peptide (KTDP) with Bilayer Membrane over a period of 125 microseconds. This video is based on MD simulations (see main text).

Movie S2. Video showing the interaction of Kinesin tail domain peptide (KTDP) with Monolayer Membrane over a period of 125 microseconds. This video is based on MD simulations (see main text).

## Data Availability

Study data are included in the article and/or supporting information.
