# Supplementary material for: Selective targeting of kinesin on lipid droplets in the liver reduces plasma lipids
Source: Proc Natl Acad Sci U S A. 2026 May 12;123(20):e2528332123. doi: 10.1073/pnas.2528332123 (PMC13187734; doi:10.1073/pnas.2528332123)
Supplement: Supplementary file 1 — Appendix 01 (PDF) [file pnas.2528332123.sapp.pdf]

SUPPLEMENTARY FIGURES, SUPPLEMENTARY INFORMATION AND SUPPLEMENTARY MOVIE CAPTIONS

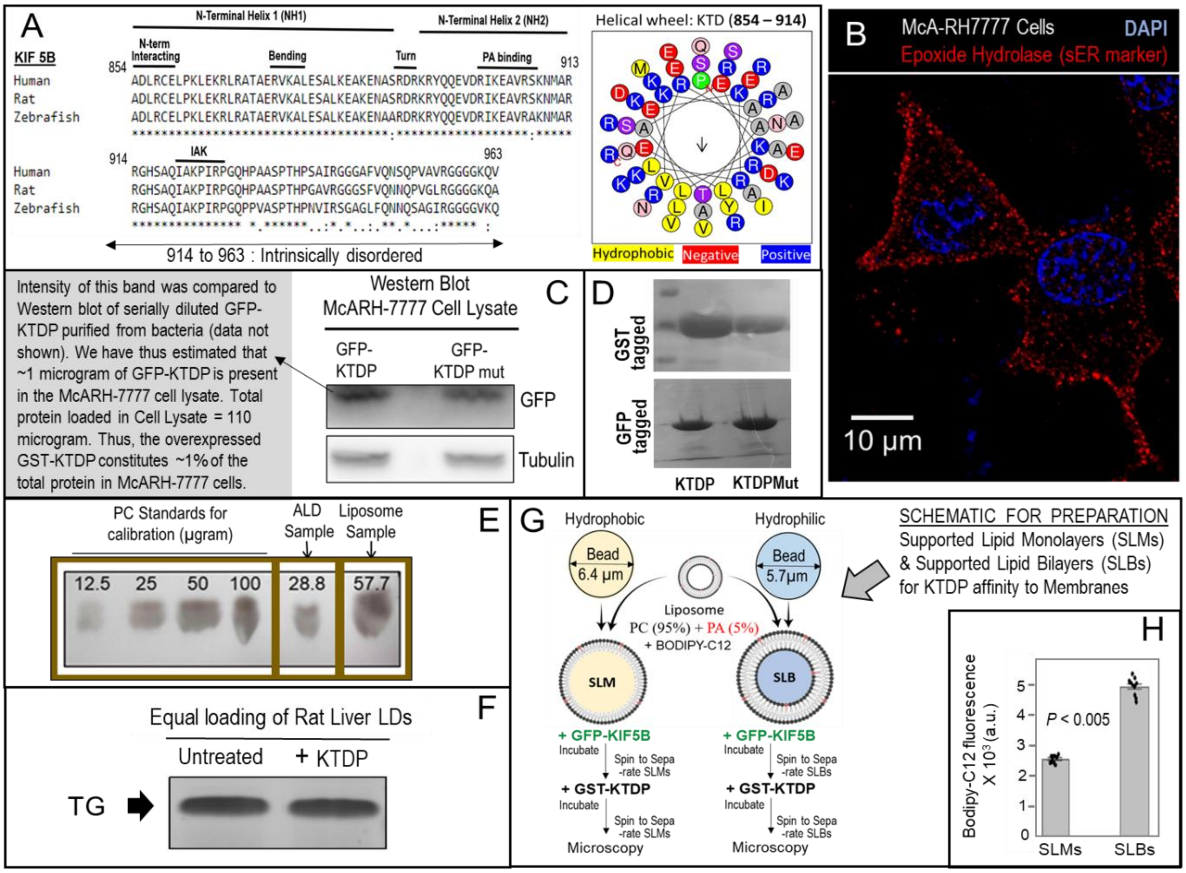

SUPPLEMENTARY FIGURE S1

Details related to KTDP, Controls for *In-vitro* experiments and Schematic of SLM and SLB Experiments.

A. LEFT PANEL :- Clustal multiple sequence alignment of KTD from KIF5B (Kinesin-1) across Human, Rat and Zebrafish species. High conservation is seen in the 854-913 AA region that has coiled coil propensity and includes the PA-binding region (901-909). RIGHT PANEL:- Helical wheel alignment of KTD AA 854-914 shows separation of hydrophobic and charged residues to opposite faces of the helix, suggesting an ability to form amphipathic helices upon membrane interaction.
B. Peripheral localization of the smooth-ER, as detected using Epoxide hydrolase antibody in McA-RH7777 cells.
C. Western blot of GFP-KTDP and GFP-KTDP-Mutant after overexpression in McARH-7777 cells. This experiment was done to estimate the amount of GFP-KTDP in cell lysate (see embedded text in figure).
D. Expression of GFP and GST-tagged KTDP and KTDP-Mutant in bacterial systems.
E. Liposomes and ALDs were prepared using (PC 95% + PA 5%). A thin layer chromatograph (TLC) was run using known dilutions of PC along with the liposome and ALD samples. The estimated amount of PC in liposome sample and ALD sample (as obtained using the PC standards) is mentioned. These values of PC were used to normalize the liposome and ALD samples, considering that 95% of both ALD and liposome membranes consists of PC (see main text).
F. Thin layer chromatograph (TLC) to detect TG shows equal loading of LDs (purified from rat liver) that were left untreated, or were treated with KTDP.
G. Schematic to explain the supported lipid monolayer (SLM) and supported lipid bilayer (SLB) assays for determining affinity of KTDP to monolayer and bilayer membranes.
H. Twice the amount of BODIPY fluorescence is detected on SLBs (bilayer) as compared to SLMs (monolayer). Each data point represents the integrated fluorescence measured along a circular profile around individual SLMs or SLBs. Errors are SEM.

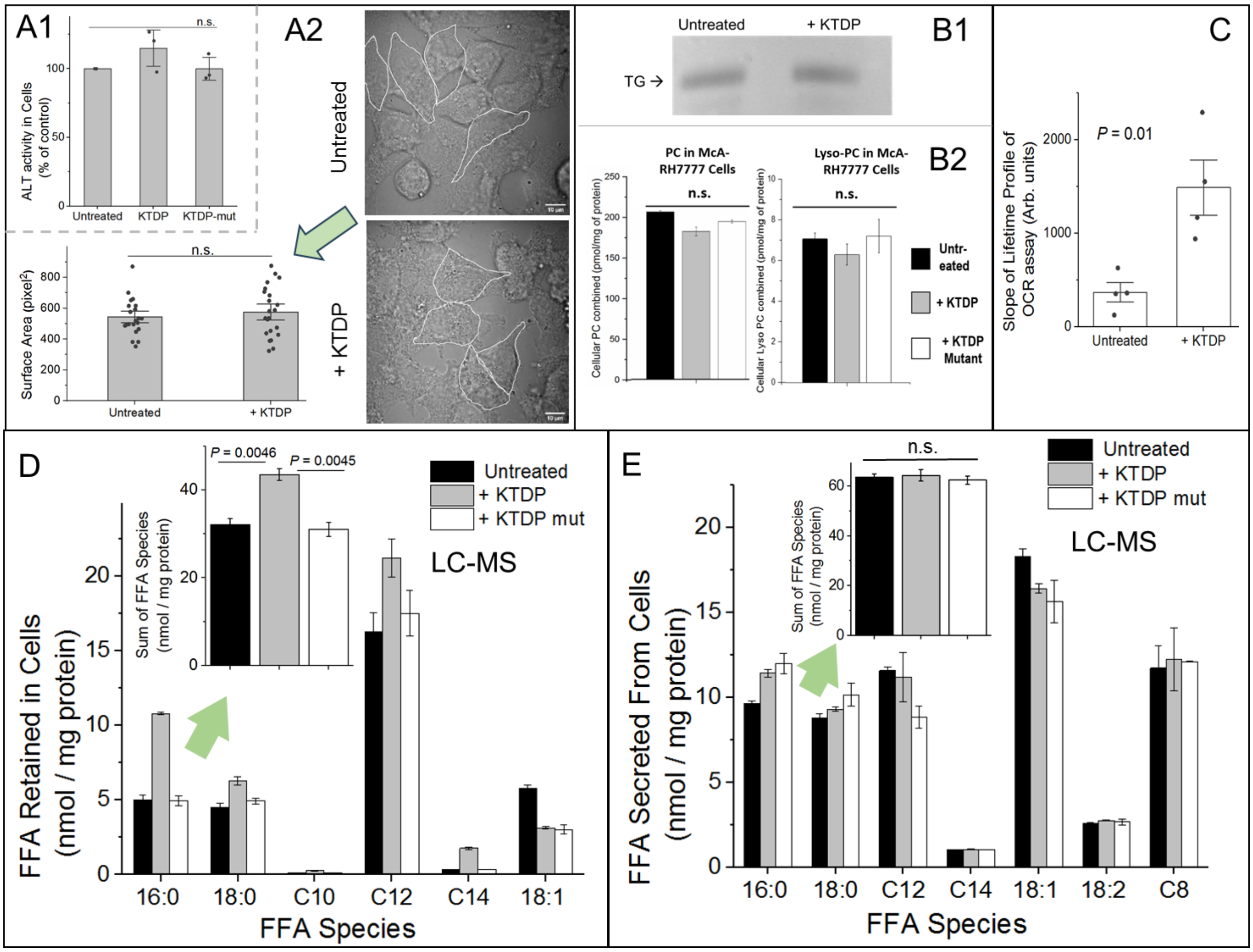

SUPPLEMENTARY FIGURE S2

ALT activity, LD biogenesis, OCR and FFA measurements using Mc-ARH 7777 Cells

A. ***(A1)*** ALT activity in McA-RH 7777 cells that are untreated, overexpressing KTDP or KTDP-mutant. ***(A2)*** Representative images of cells (outlines shown as a visual guide) with cell surface areas measured from such images across untreated and KTDP treated conditions. Scale bar = 10 microns. Data are mean ± SEM; ns = not significant.

B. ***(B1)*** TLC of McA-RH 7777 cells that are untreated or overexpressing KTDP. Cells were first depleted of LDs by keeping in serum-free medium followed by addition of Oleic acid to induce LD biogenesis for 12 hours. Cells were then lysed, and TLC of cell lysate was run. The TG band is similar across conditions suggesting no effect of KTDP on LD biogenesis. ***(B2)*** LC-MS measurement shows that KTDP caused no significant change in a major cellular phospholipid (PC) and Lyso-PC.

C. Cellular oxygen consumption rate (OCR) showing increased cellular respiration in KTDP-overexpressing McA-RH 7777 cells compared to untreated. Data are mean ± SEM.

D. LC-MS measurement of free fatty acids (FFAs) retained in Mc-ARH 7777 cells. The sum of detected FFA species is also shown (errors have been propagated). There is a significant increase of FFAs inside cells after overexpression of KTDP, suggesting the activation of a lipolytic pathway. Errors are SEM.

E. LC-MS measurement of FFA species secreted from Mc-ARH 7777 cells. The sum of detected FFA species is also shown (errors have been propagated). There is no significant effect of overexpressing KTDP on FFA secretion from cells. Errors are SEM.

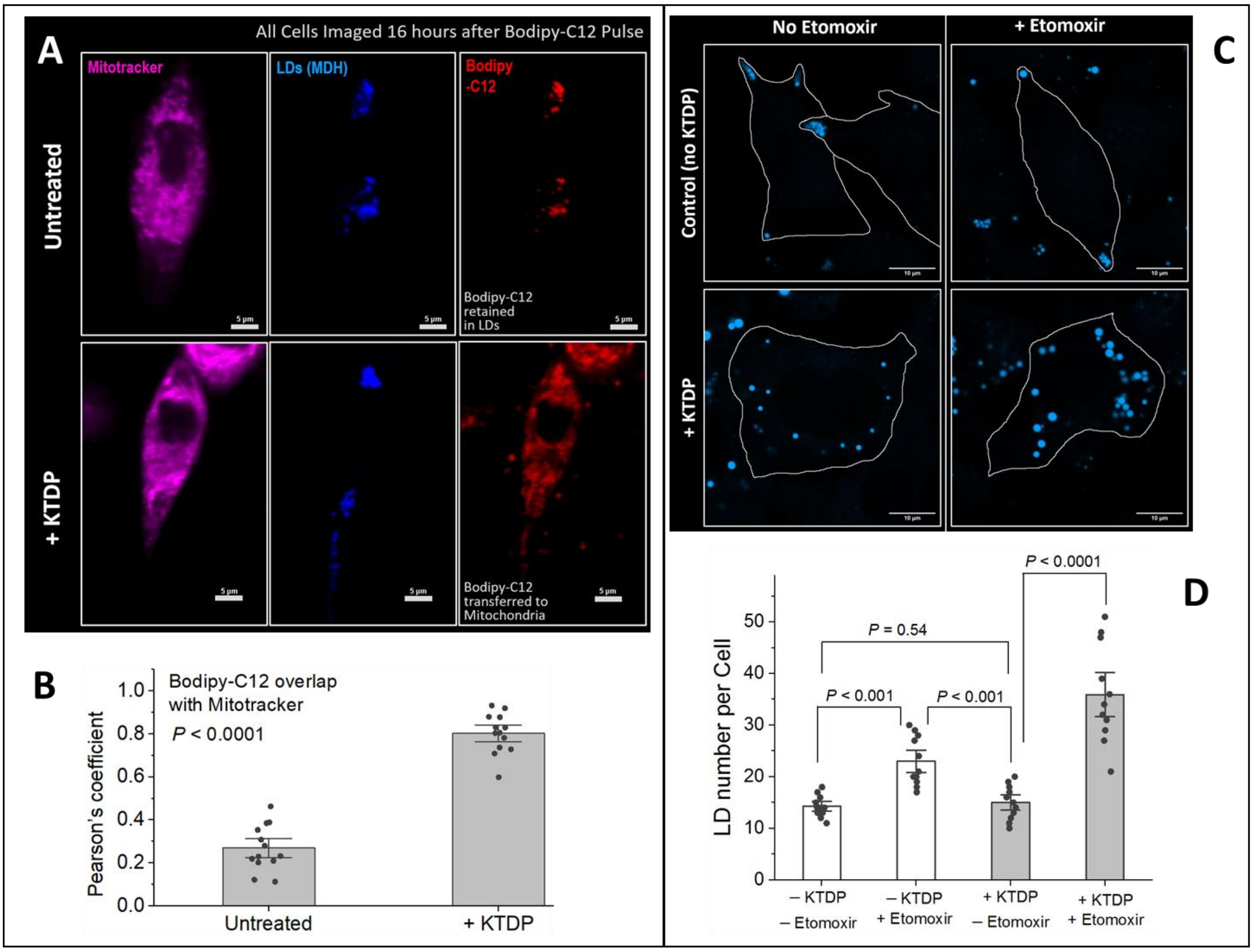

SUPPLEMENTARY FIGURE S3

A. Intracellular fatty acid trafficking in untreated (control) and KTDP-overexpressing McA-RH7777 cells that were subjected to a pulse with BODIPY-C12 Red followed by a 16-h chase before imaging. Mitochondria were stained with MitoTracker Deep Red and LDs were visualized using MDH dye. Scale bar = 5 µm.

B. Colocalization of BODIPY-C12 with MitoTracker was quantified using Pearson's correlation coefficient analysis. Each data point represents result from an individual cell. Data are presented as mean ± SEM.

C. Confocal images of McA-RH7777 cells (without or overexpressing KTDP) that were either left untreated or treated overnight with etomoxir. Etomoxir blocks mitochondrial fatty acid import by inhibiting carnitine palmitoyltransferase-1 (CPT-1). LDs were imaged using MDH (blue) neutral lipid dye. Note how control cells have LDs at cell periphery, but KTDP causes LD re-distribution to all parts of the cells. Scale bar = 10 µm.

D. Quantification of LD numbers. Control cells (no KTDP over-expression; white bars) exhibited ~1.5-fold increase in LD numbers after etomoxir, likely reflecting basal inhibition of fatty acid oxidation. For KTDP treated cells (gray bars), etomoxir resulted in a significantly higher increase (~2.5-fold) of LD numbers, suggesting that KTDP induced enhanced lipid trafficking from LDs to mitochondria. This possibility is also supported by observing LDs of the largest size in KTDP-expressing etomoxir treated cells. Data are Mean ± SEM.

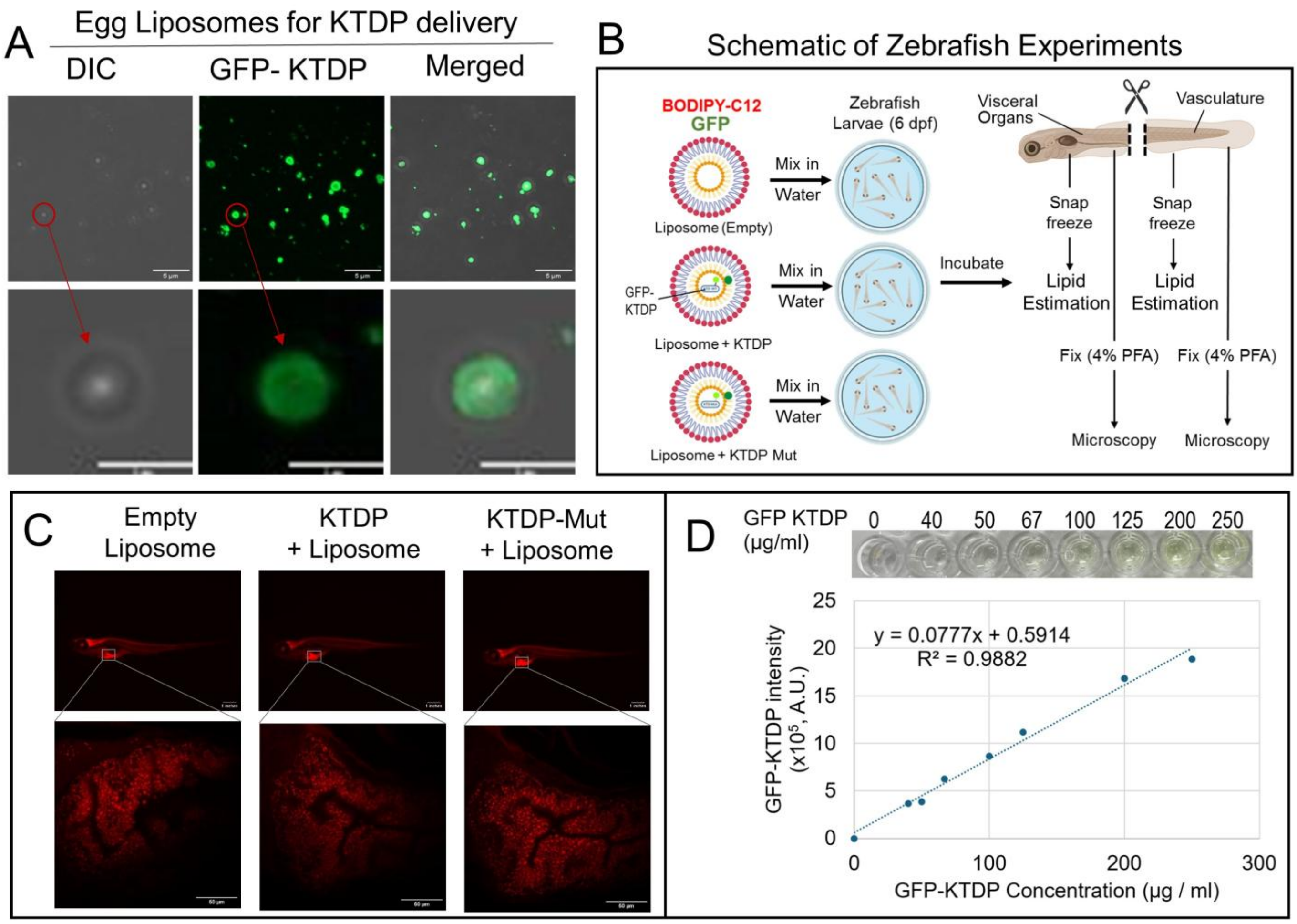

SUPPLEMENTARY FIGURE S4

Experiments related to Egg-Liposomal Delivery KTDP in Zebrafish Larvae

A. Microscopy of Egg liposomes containing GFP-KTD. DIC (Differential Interference Contrast) and Confocal images are shown. GFP signal is clearly visible within nearly all liposomal vesicles. Scale bar = 5 µm.
B. Schematic representation of the feeding protocol in which BODIPY-C12-liposomes (with and without KTDP) are administered to 6 days post-fertilization (dpf) zebrafish larvae, followed by microscopy and biochemical analysis to observe the effect of KTDP on lipid secretion.
C. Confocal images showing BODIPY fluorescence (red) in zebrafish larvae (6 dpf) and in the gut of larvae after incubation with BODIPY-containing liposomes across conditions (mentioned). Results indicate equal feeding across experimental groups.
D. Fluorescence intensity gradient of GFP-KTDP corresponding to increasing protein concentrations. Standard curve showing the linear relationship between fluorescence intensity and protein concentration of purified GFP-KTDP.

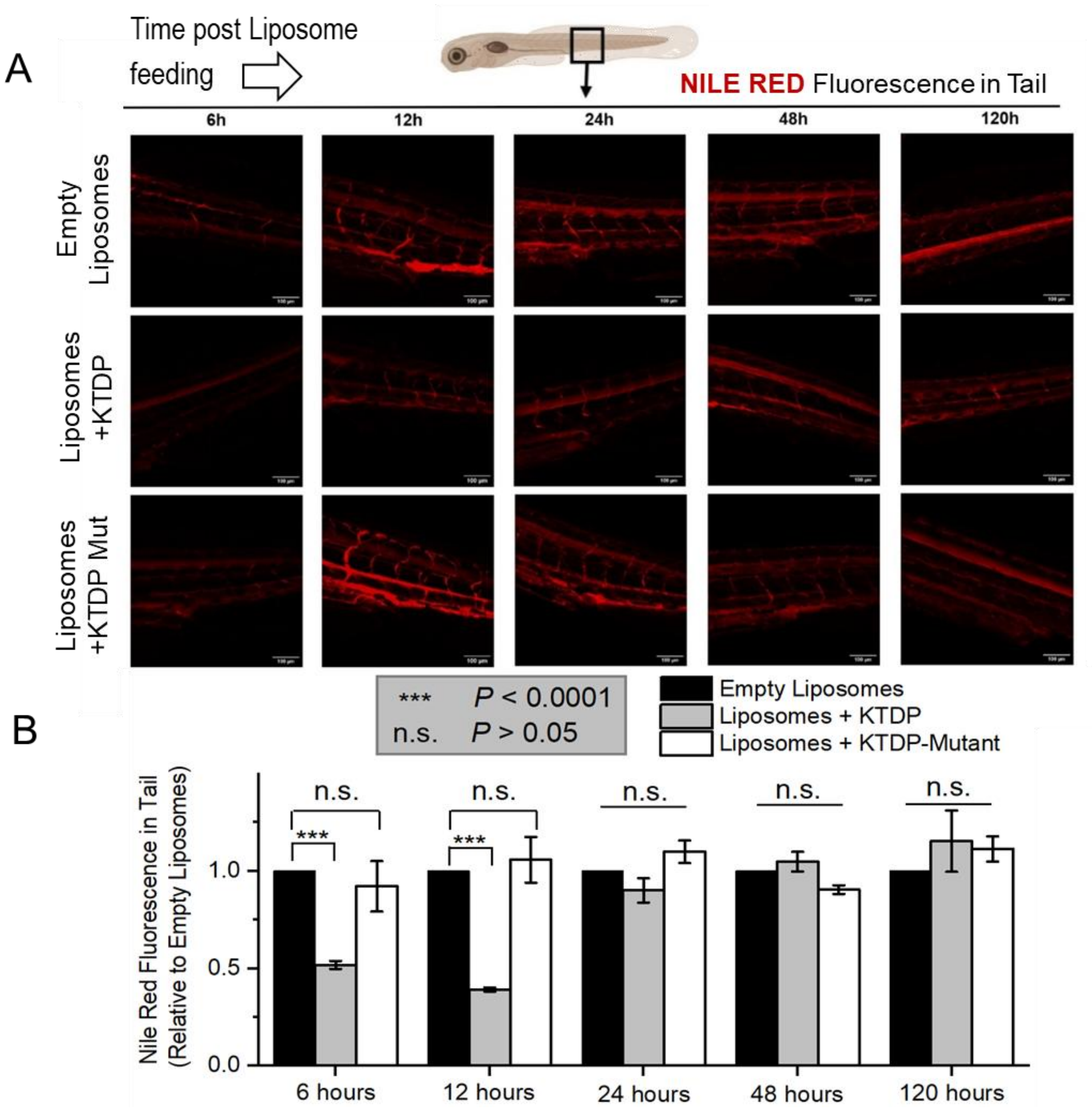

SUPPLEMENTARY FIGURE S5

Time course of Lipids in Tail of Zebrafish Larvae after Liposomal feeding of KTDP.

A. Representative confocal images of Tail region (vasculature) of Nile red stained Zebrafish larvae at 6, 12, 24, 48 and 120 hours post incubation with Egg Liposomes (unlabelled) that were Empty, containing KTDP or KTDP-mutant.
B. Fluorescent intensity in larval tail, expressed as fold change relative to larvae treated with Empty liposomes at that time-point. The data reveal ~50% reduction of lipid content in the tail at 6 and 12 hours post administration of Liposomes, followed by reversal to baseline levels. Overall fluorescence intensity at the 6 hour time point is lower because the lipids contained in egg-liposomes have presumably not yet been metabolized and secreted out into vasculature. Each bar represents the average fluorescence intensity measured in 3 larvae ($N = 3$). Error bars are SEM. *** denotes $P \leq 0.0001$, n.s. implies $P > 0.05$.

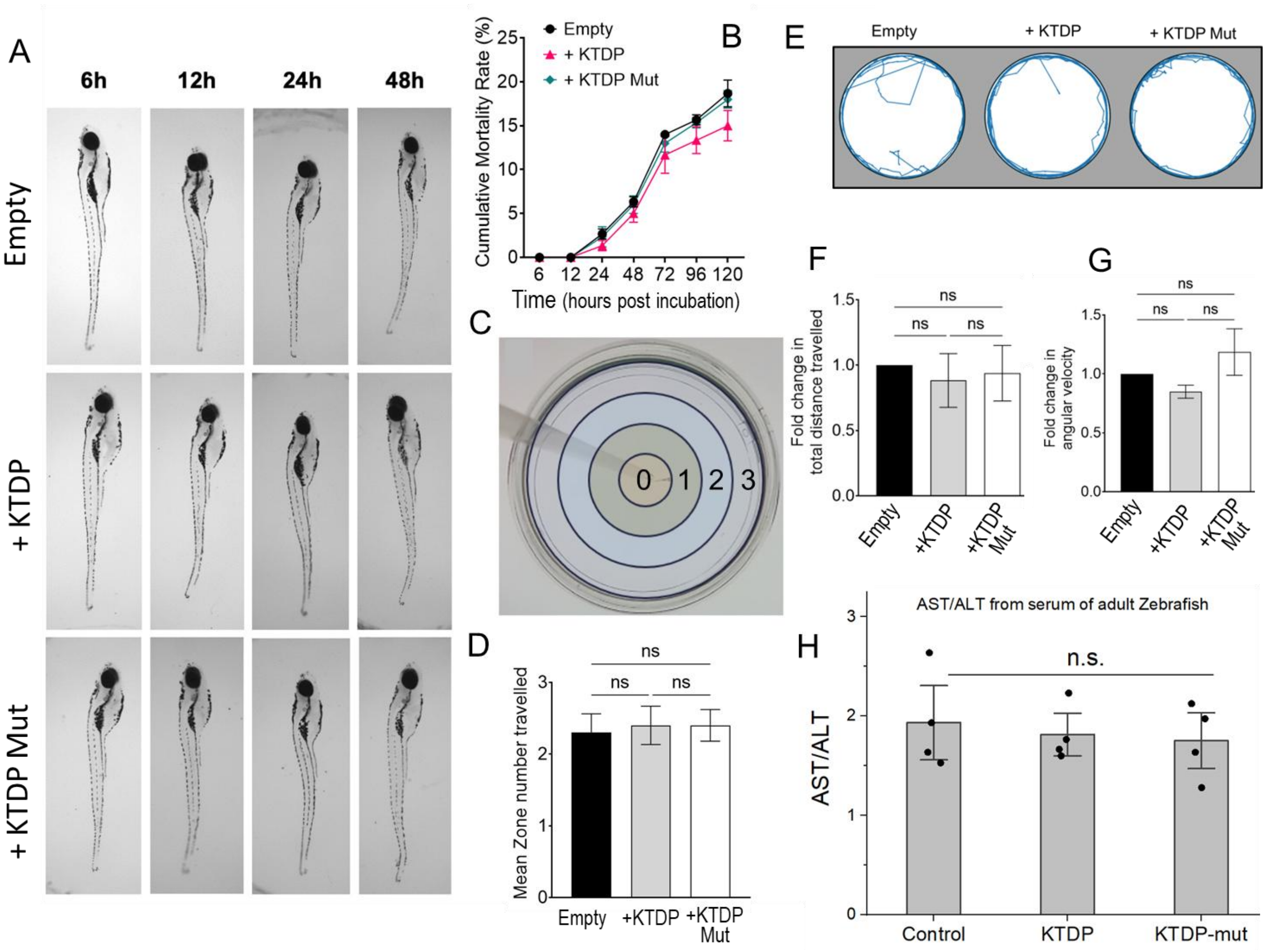

SUPPLEMENTARY FIGURE S6

Effect of KTDP on Morphology, Mortality and Locomotion of Zebrafish Larvae

A. Images of Zebrafish larvae at 6, 12, 24, and 48 hours post-incubation with egg-liposomes that were Empty, containing KTDP or KTDP-Mut. No observable effect of KTDP is seen on the morphology of larvae.
B. Mortality rates of larvae were monitored up to 120 hours post-incubation with egg-liposomes that were Empty, containing KTDP or KTDP-Mut. No significant differences are seen across experimental groups.
C. Petri dish setup illustrating concentric zones for studying larval locomotion. Individual larvae were placed at the center and stimulated with a micropipette tip. The zone where individual larvae first stopped after stimulation was noted. Zones were defined concentrically, with the center being Zone 0 and the outermost being Zone 3.
D. Results of micropipette stimulation indicate no significant differences in the first stop zones among experimental groups at 120 hours post KTDP feeding, suggesting no impairment in touch-evoked response (TER).
E. Trajectories of individual larvae recorded over six minutes in a six-well plate that were analyzed using ZebraZoom software.
F. Fold change in total distance traveled by larvae from video recordings.
G. Fold change in angular velocity derived from the same video recordings. These results indicate no significant change in locomotory behavior after incubation with KTDP.
H. AST/ALT ratio in the serum of adult Zebrafish across untreated (control), KTDP-fed and KTDP-mutant fed conditions. No significant difference is observed across conditions.

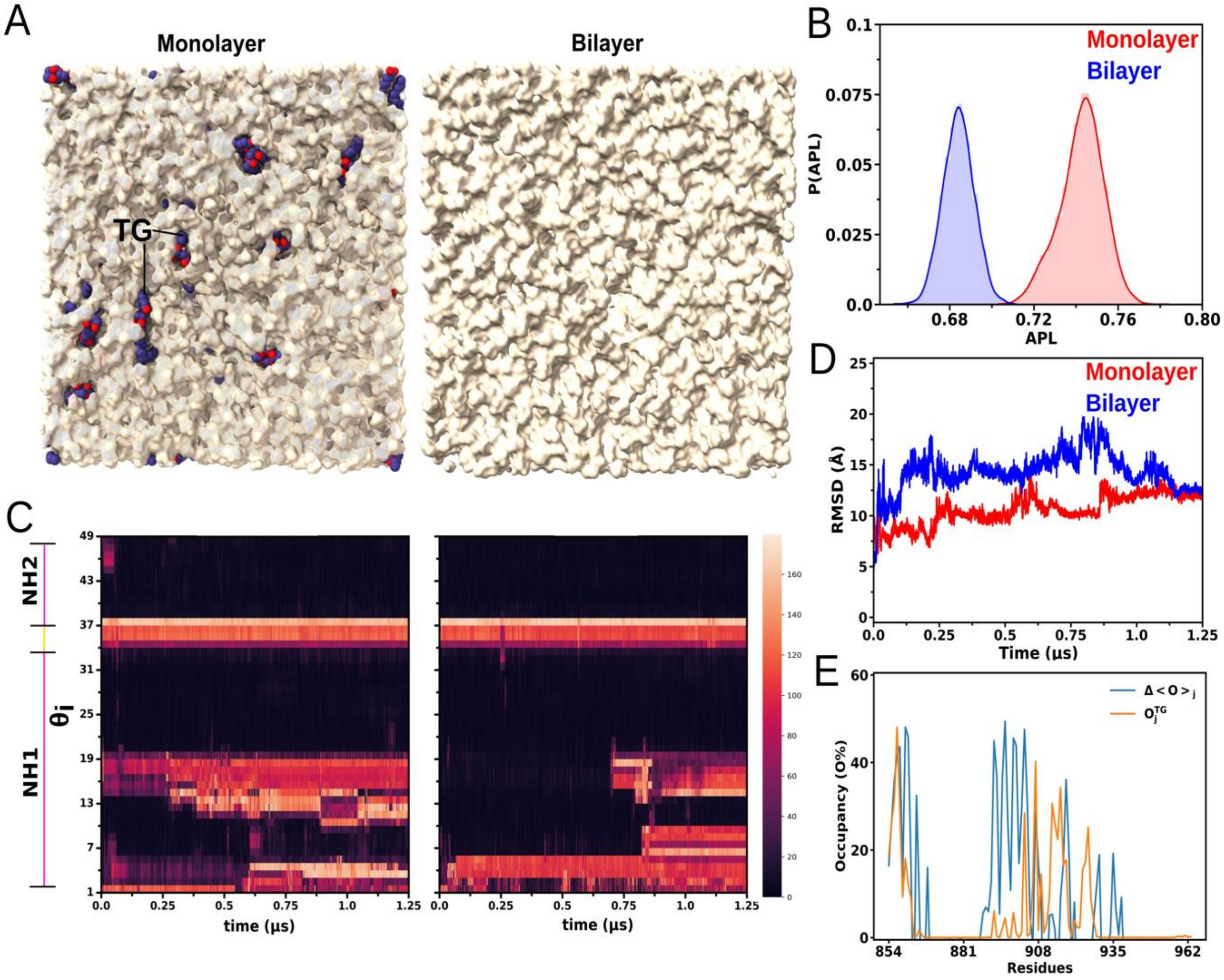

SUPPLEMENTARY FIGURE S7

Additional Details for MD Simulations of KTDP interacting with Monolayer and Bilayer Membranes

A. A top view representation of membrane leaflets of monolayer and bilayer systems. Surface-exposed TG molecules (dark purple) are observed only on the monolayer.
B. Probability distributions of the area per lipid (APL, in nm^2) on monolayer and bilayer systems.
C. Helix distortion of KTDP on the monolayer and bilayer systems as a function of time (see main text). The KTDP N-terminal helices (NH1 and NH2) are indicated.
D. Backbone RMSD of KTDP in monolayer and bilayer over the simulation trajectory.
E. Monolayer favouring average lipid occupancy $(\Delta< O >_{ij})$ > 0 and TG occupancy (O_j^{TG}) during KTDP-monolayer interactions. Note the correlation between TG and PC/PA interactions, suggesting that exposed TG patches on the monolayer (see panel A) are important for these interactions.

MATERIALS AND METHODS, *Tripathy et. al.*

Note :- Some References in Supporting Information have a Separate Reference List (Indicated wherever relevant)

Reagents Used

GFP-KLC1E plasmid-a generous gift from Prof. mark Dodding, University of Bristol. DMEM (Sigma-D7777), DMEM no glucose (Gibco-11966025) FBS (Gibco-16000-044), trypsin-EDTA (15400054), Pen Strep (Gibco-15070063), Lipofectamine 2000 (Invitrogen-11668019), OPTI MEM (Gibco-31985070), Fatty acid free BSA (Sigma- A8806), BSA (Genei- 1650500501730), Monodansylpentane (MDH; abcepta-SM1000a), LipidTOX (Invitrogen- H34476), Silica gel 60 plates (Merck Milipore-1.05721.0001), Protease inhibitor cocktail (Roche-13539320), Ni-NTA sepharose resin (Cytiva-17531801), GST sepharose beads (Qiagen, 27-4574-01), egg PC (Avanti-840051P), egg PA (Sigma-P9511), Glycerol trioleate (Sigma- T7140), BODIPY C12 (Invitrogen-3822), Alexa 647 dye (Invitrogen-A20173), Chemiluminescent HRP substrate (Milipore-WBKLS0500), Latex beads, styrene divinylbenzene (Sigma-SD6A), Latex beads carboxylate (Polysciences-17141), Nile red (Sigma-72485), ALT assay kit (Cayman Chemical – 700260), TG assay kit (Elabscience - E-BC-K238), Cholesterol assay kit (Elabscience - E-BC-K109-S), Extracellular oxygen consumption assay kit (abcam-ab197243), Antibodies for Lamp1 (abcam-ab24170), KDEL (abcam-ab12223), perilipin-2 (Progen-651102), GFP (Invitrogen-A11122), α-tubulin antibody (Sigma-T9026), GST (Cloud clone-TAX158Ge22), secondary Alexa Fluor 555 donkey anti-rabbit (Thermo Fisher-A31572), donkey anti-rabbit IgG-HRP (Santa cruz-sc2313) and donkey anti-mouse IgG-HRP (Santa cruz-sc2314), Kinesin 1 (custom made). Mitotracker deep red (Invitrogen M22426), Etomoxir (Med Chem Express – HY 50202).

Animal strains and Procedures

All zebrafish (*Danio rerio*) care and experimental procedures were conducted in accordance with guidelines approved by the Institutional Animal Ethics Committee of IIT Bombay (Approval no. IITB/2025/BSBE/RM01). Zebrafish husbandry, breeding, and maintenance followed established standard protocols. Adult zebrafish, encompassing wild-type (Tübingen) and relevant transgenic lines [Tg(fabp10a:DsRed)], were maintained in a recirculating system with controlled water parameters [temperature (e.g., 28 ± 0.5°C), pH (e.g., 7.2-7.6), conductivity (e.g., 500-600 µS/cm)] under a 14-hour light / 10-hour dark photoperiod. Natural spawning was induced by placing male and female fish (typically in a 1:1 or 2:1 ratio) the evening prior into breeding tanks equipped with dividers. Dividers were removed the following morning upon commencement of the light cycle, and embryos were typically collected within 1-2 hours post-fertilization. Collected embryos were rinsed and subsequently reared in standard E3 embryo medium (5 mM NaCl, 0.17 mM KCl, 0.33 mM $CaCl_2$, 0.33 mM $MgSO_4$, buffered with 7.5 mM sodium bicarbonate to pH 7.3 and containing 0.0001% methylene blue) at ~50 embryos in a 100 mm Petri dish in an incubator maintaining a constant temperature of 28.0 ± 0.5°C and the same 14:10 light:dark cycle. The E3 medium was refreshed daily to maintain water quality. Larvae were fed starting from 6 dpf, the feeding regimen consisted of a combination of dry feed and live artemia culture administered thrice daily. For procedures requiring immobilization such as imaging or fixing, larvae were anesthetized using 0.06% Tricaine.

Sprague-Dawley rats were bred and maintained by the animal house facility at the Tata Institute of Fundamental Research, Mumbai and animal protocols approved by the Institutional Animal Ethics Committee. Rats were maintained on a regular light (12-h)/dark (12-h) cycle and fed a standard laboratory diet. 8–12 week old male Sprague-Dawley rats were used for all the experiments.

Cell lines and Growth Conditions

HEK-293T (ATCC CRL-11268) and McA-RH7777 (ATCC CRL-1601) cell lines were used. HEK-293T cells were cultured in DMEM supplemented with 10% fetal bovine serum (FBS). McA-RH7777 cells were grown in DMEM containing 20% FBS. All cells were maintained at 37 °C in a humidified incubator with 5% CO_2. Cells were subcultured using trypsin-EDTA upon reaching 70-80% confluency.

LD and Lysosome Distribution in McA-RH 7777 Cells

McARH-7777 cells were seeded on acid-wash coverslips and allowed to grow till 50%-60% confluency. The cells were transfected with GFP-KLC, GFP-KTDP and GFP-KTDP Mutant plasmids using Lipofectamine 2000 (Thermo Fisher Scientific). Briefly, Plasmid DNA and Lipofectamine 2000 were separately diluted in Opti-MEM and Lipofectamine were mixed with Opti-MEM minimal media (Gibco) and then combined and incubated for 20 minutes at room temperature to generate transfection complexes. Control cells were treated only with lipofectamine, but no plasmid. The complexes were added to the cells and incubated for 6 hours followed by replacing the media with FBS (20%) containing high glucose DMEM media. Cells were grown for 48 h in the incubator and then treated with 400 µM BSA-conjugated OA for LD induction and cultured at standard conditions for 6 h. Cells were fixed with 4% PFA for 15 minutes and washed thrice with 1× PBS followed by permeabilization with 0.1% Triton X-100 in PBS for 10 minutes. The cells were again washed with PBS and blocked with 5% BSA in PBS for 30 minutes at room temperature. For lysosomal staining, cells were treated with primary LAMP1 antibody (1:100 dilution) for 1 hour at room temperature in a humidified chamber followed by washing thrice with PBS. The respective secondary antibodies (dilution 1:500) were added and incubated at room temperature for 1 h. The unbound antibodies were removed by washing with 1X PBS thrice. Cells were mounted with mounting medium and 50 µM monodansylpentane (MDH) for lipid droplet visualization. Fluorescence images were captured using a ZEISS LSM 900 confocal microscope with a 63× oil immersion objective. Image analysis was done using ImageJ-Fiji.

LD distribution was quantified by calculating the relative position of each LD within the cell (7). Lysosomal distribution was assessed by analyzing the spatial localization of LAMP1 fluorescence within the cell as discussed in (19). Fluorescence intensity

in the peripheral region of the cell was measured and normalized to the total LAMP1 fluorescence intensity across the entire cell. The resulting ratio provides a measure of lysosomal distribution, with higher values indicating increased peripheral localization.

Bioinformatics Analysis of KTDP:
Secondary structure prediction, Multiple sequence alignment, Helical wheel diagram.

Sequence alignment of KTDP across humans, rat and zebrafish was performed to assess evolutionary conservation. The analysis demonstrated a high degree of sequence identity with 94% similarity observed between human and rat KTDP, and 83% between human and zebrafish. The helical region critical for membrane binding (amino acids 854–913) exhibited strong conservation across all three species. To investigate the helicity nature of the N-terminal region of KTDP, a helical wheel projection was generated for amino acids 854-914 using HeliQuest web server (https://heliquest.ipmc.cnrs.fr/). The sequence was input in single-letter amino acid format, and an α-helical conformation was assumed.

Expression and Purification of GST-KTDP and GFP-KTDP from Bacteria

GST tagged fusion proteins were expressed in *E. coli* (BL21DE3) and purified using glutathione-conjugated sepharose beads using the manufacturer's protocol. Briefly, BL21DE3 competent cells were transformed with pGEX-4T1 plasmid containing KTDP and KTDPmut fusion protein gene. A single colony was inoculated for primary culture at 37 ^{o}C overnight. Secondary culture (1% inoculum) was induced with 1 mM IPTG at OD600 = 0.6 and incubated overnight at 18°C with shaking. Cells were harvested by centrifuging the culture at 5000 g for 30 minutes and the pellet was resuspended in lysis buffer (50 mM TRIS-HCl, 150 mM NaCl, 5 mM beta-mercaptoethanol, 0.1% triton, 1 mM PMSF, 1 mg/mL lysozyme and 1X PIC, pH = 8.0) followed by lysis using probe sonication (amplitude=30%, 12 cycles, each of 15s ON and 45s OFF). Lysate was centrifuged at 20,000 rpm for 1 hour at 4°C and the supernatant was incubated with Glutathione Sepharose Beads pre-equilibrated with lysis buffer for 3 hours at 4 ^{o}C on a nutator. Beads were washed with 10 column volumes of wash buffer (50 mM TRIS-HCl, 150 mM NaCl, 5 mM β-mercaptoethanol, 1 mM PMSF, pH = 8.0) and bound proteins were eluted with reduced glutathione buffer (50 mM TRIS-HCl, 150 mM NaCl, 5 mM β-mercaptoethanol, 40 mM reduced glutathione, pH = 8.0). Eluate was dialyzed overnight against 1×PBS (pH = 7.4). Protein purity was assessed by 12% SDS-PAGE followed by Coomassie Brilliant Blue staining or anti-GST immunoblotting. Protein concentration was measured using the Bradford assay.

GFP-KTDP-His proteins were expressed in *E. coli* BL21(DE3) using 1 mM IPTG induction at OD600 = 0.6 and incubation at 18°C, overnight. Cells were harvested by centrifugation followed by lysis using sonication in lysis buffer (50 mM sodium phosphate, 300 mM NaCl, 0.1% Triton X-100, 5 mM β-mercaptoethanol, 1× PIC, pH 8.0) and centrifuged (20,000 g, 1 h, 4°C). Supernatant was bound to Ni-NTA resin for 3 hours at 4^{0}C on a nutator and washing was done with washing buffer (50 mM sodium phosphate, 300 mM NaCl, 50 mM imidazole). Protein were eluted with 300 mM imidazole in 50 mM sodium phosphate, 300 mM NaCl, 1X PIC. Eluted protein was dialyzed into 1X PBS and analyzed by SDS-PAGE/Coomassie.

Preparation of Artificial Lipid Droplets (ALDs) and Liposomes

ALDs were prepared by using a freeze-thaw technique (7). Briefly, 0.5 µmol egg phosphatidylcholine (PC) and 25 nmol of egg phosphatidic acid (PA) were mixed in clean glass tubes to prepare 1 ml of ALDs. This reaction mixture was dried under nitrogen gas stream for 30 minutes. Then 70 µl of glycerol trioleate was added in the dried glass tube followed by vacuum desiccation for 3-6 h to remove trace amounts of chloroform. After desiccation, 930 µl HKM buffer (50 mM HEPES-KOH, 120 mM potassium acetate, and 1 mM $MgCl_2$, at pH 7.4) was added to the dried reaction mix for hydration. This reaction mixture was vigorously mixed by vortexing for 10 minutes to aid emulsification. The whitish emulsion was poured into ultra-low temperature resistant tubes and flash frozen in liquid nitrogen. The frozen emulsion was immediately thawed in a water bath preset at 55°C. This freeze–thaw process was repeated for five cycles with intermittent vortexing before every freezing step.

Liposomes were prepared by using a standard freeze-thaw technique (42). 1 µmol egg PC with 50 nmol of Egg-PA were dried in clean glass tubes and the reaction mixture was dried under nitrogen gas stream for 30 minutes followed by vacuum desiccation for 3-6 h to remove trace amounts of chloroform. The dried lipid film was hydrated with 1 ml HKM buffer (50 mM HEPES-KOH, 120 mM potassium acetate, and 1 mM $MgCl_2$, at pH 7.4) and vortexed vigorously for 10 minutes. The mixture was subjected to five cycles of flash freezing in liquid N_2 and thawing at 55°C. Each thawing step was followed by vigorous mixing to enhance unilamellar vesicle formation. For labelling the liposomes, BODIPY-C12 (8 µM) was added to the liposomes, mixed well and kept undisturbed at RT for 30 minutes.

Normalization of ALDs and Liposome Samples by Thin Layer Chromatography (TLC)

1 ml ALDs and 1ml liposomes were taken in two separate glass tubes. 2 ml methanol and 1 ml chloroform (2:1 v/v) were added to each tube followed by vigorous vortexing. The glass tubes were kept overnight at 4˚C. The next day, 1 ml chloroform and water (1:1 v/v) were added to the tubes, vortexed, and kept undisturbed to allow phase separation under gravity (centrifuged at 1000 g for 10 minutes) to obtain a clear lower organic phase having phospholipids. Equal volumes of the lower organic phase were collected using Hamilton syringe and transferred to a new glass tube followed by drying under a stream of nitrogen gas (ReactiVap) for approximately 30 minutes. After drying, the phospholipid mix was resuspended in 50 ul of chloroform and spotted using a glass capillary on 8 X 10 cm a Silica TLC plate (Merck) that has been pre-rinsed with chloroform and dried overnight. Phosphatidylcholine standards of amounts 12.5, 25, 50, 100 µg were loaded to generate a calibration curve. Phospholipids were resolved by using Chloroform: Methanol: Ammonium hydroxide (65:25:4) as solvent system and was run till the solvent front reached approximately 95% of the plate *(See Ref. 1 in SI Reference List)*. The plate was air dried and the bands were visualized by spraying 10% $CuSO_4$ in 8% H_3PO_4 followed by baking the plate in the oven at 100^{0}C for 30 minutes. The plates were scanned and the bands were quantified using ImageJ. A calibration curve was generated using known phospholipid amounts to correlate band intensity with concentration. ALD and liposome phospholipid content was interpolated from the curve and normalized to surface area.

KTDP Interaction with ALDs and Liposomes

Normalized volumes (ALD:liposome = 2:1 v/v) of ALDs and liposomes were taken in two tubes. Equal amount (5 µM) of GST-KTDP protein were added to both the tubes and incubated on nutator at RT for 2 h. Both ALDs and liposomes were isolated from unbound proteins by density gradient ultracentrifugation using MEPS (5mM MgSO4, 5mM EGTA, 35mM PIPES pH=7.2, sucrose) buffer. The reaction mixture containing ALDs was supplemented with 1.5 times vol/vol of 2.5 M sucrose containing MEPS buffer and was loaded at the bottom layer of sucrose density gradient. This layer was overlaid with 5 ml (each) of 1.2M, 0.5M, and 0M sucrose in MEPS buffer. The gradient was centrifuged at 120000g using SW32 rotor at 4°C for 1 h to obtain lipid droplets (top-most whitish layer). Liposomes were pelleted at 100000g (transparent pellet). ALDs were collected using an 18-G needle and concentrated by centrifuging again at table top centrifuge at 20,000 g for 10 minutes. Liposome pellet was also resuspended in the same volume as concentrated LDs in 1X PBS.

Protein Precipitation and Western blotting

Equal volumes of ALDs or liposomes were mixed with chloroform:acetone (1:1 v/v) in two different safe lock tubes. The tubes are vortexed thoroughly and stored at -20˚C overnight. The next day the tubes are vortexed vigorously and stored at -20˚C for 15 minutes. Precipitated proteins were collected by centrifugation at 20,000 g for 2 h. The pellet was solubilized in 2× sample buffer and kept at 95°C for 15 minutes. The samples were loaded on 10% SDS-PAGE.

For Western blotting, the proteins on the SDS-PAGE gel were transferred to a PVDF membrane. The membrane was blocked with 5% non-fat dry milk in Tris-buffered saline with 0.1% Tween-20 (TBST) for 1 h at RT. The membrane was incubated with primary anti-GST antibody diluted in 5% BSA for overnight at 4°C. The membrane was washed thrice with TBST for 10 minutes each. The membrane was incubated with HRP-conjugated secondary antibody diluted in blocking buffer for 1 h at RT. The membrane was washed with TBST three times for 10 minutes each and developed using ECL. The blots were imaged on a ChemiDoc, and band intensity was quantified using ImageJ.

Isolation of Microsomes and Lipid Droplets from Rat Liver

Endoplasmic reticulum enriched microsomes were isolated using the protocol described in (42). Rats were anesthetized using sodium thiopentane (40 mg/kg body weight). The abdomen was cut open, and the liver was perfused with 50 mL of cold PBS (1× PBS) through the hepatic portal vein and then isolated. Liver tissue (9 gm) was minced in cold room with 3 volumes of 0.25 M MEPS buffer containing 4 mM DTT, 8 µg/µL pepstatin, 4 mM PMSF, and Roche protease inhibitors. The minced tissue was then homogenized on ice in a 50-mL Potter-Elvehjem grinder using a ribbed Teflon pestle (up to 20 strokes). The homogenate was filtered through a two-layered cotton mesh and then centrifuged (8,700g, 4°C, 15 minutes) to obtain the postnuclear supernatant (PNS). The PNS was centrifuged (43,000g, 4°C, 7 minutes) to pellet mitochondria followed by centrifuging the supernatant (110,000g, 4°C, 60 minutes) to isolate the microsomes. Microsome pellet was resuspended in 1× PBS, flash-frozen, and stored at −80 °C for further experiments.

LDs were isolated from rat liver using the previously described protocol (7). Male Sprague–Dawley rats (3-4 months old) were anesthetized with sodium thiopentone (40 mg/kg, i.p.), liver was perfused with cold PBS via portal vein, excised, minced and homogenized in 1.5× (w/v) 0.9 M sucrose with MEPS buffer (with protease inhibitors) using a Dounce homogenizer at 4 °C. Homogenate was centrifuged (1800g, 10 minutes, 4 °C) to obtain the postnuclear supernatant (PNS) which was mixed with 1.5× vol of 2.5 M sucrose in MEPS buffer and layered under a step gradient of 1.2 M, 0.5 M, and 0 M sucrose in MEPS buffer. After centrifugation at 120,000g for 1 h at 4 °C, LDs (top whitish layer) were collected using an 18G needle, flash-frozen, and stored at -80 °C.

Removal of Endogenous Kinesin-1 by KTDP from LDs and Microsomes

Isolated LDs were divided into two tubes having equal volumes. One tube was treated with 5 µM GFP-KTDP while the other tube received equal volume of 1X PBS as untreated condition. Samples were incubated on a thermomixer at 37^0C for 1 hour at 500 rpm. Following incubation, unbound proteins were separated by sucrose density gradient ultracentrifugation (previously described). LDs were collected from the topmost layer and concentrated to equal volume. Treated and untreated LDs were normalized by optical density at OD_{600} (22). Equal volumes of LDs were mixed with 0.1% SDS, heated at 60 degree for 30 minutes with intermittent vortexing, and centrifuged at 20000g for 20 minutes. The aqueous middle phase was collected, mixed with 1x loading dye and loaded on SDS-PAGE.

A similar protocol was followed for microsomes. Equal volumes of microsomes in separate tubes were incubated with 5 µM GFP-KTDP (KTDP treated condition) and 1X PBS (untreated condition) at 37^0C for 1 hour with mixing at 500 rpm on a thermomixer. After incubation, unbound proteins were separated by ultracentrifugation and the microsomal pellets were resuspended with equal volumes of chilled 1X PBS. Equal volumes of treated and untreated microsomes were mixed with 1x loading dye and subjected to SDS-PAGE.

Western blotting was performed using anti-Kinesin-1 antibody to assess endogenous Kinesin-1 levels, anti-GFP antibody to detect GFP-KTDP binding, anti-Perilipin-2 as an LD marker, and anti-KDEL as an ER/microsome marker. Band intensities were quantified using ImageJ to compare endogenous Kinesin1 levels and GFP-KTDP binding between treated and untreated LDs and microsomes.

TLC for Normalization of LDs

Lipids from LDs were extracted by the methanol:chloroform method mentioned earlier. Extracted lipids were dried, resuspended in chloroform and then loaded onto silica plates pre-rinsed with chloroform. Separation was performed using two step solvent system i.e. in solvent I (n-hexane/ diethyl ether/acetic acid, 70:30:1) till half way and air dried, then in solvent II (n-hexene/diethyl ether, 59:1) for complete run. After air drying, TG bands were visualized by spraying the plate with 10% CuSO4 in 8% H3PO4, followed by baking >100 °C for 30 minutes.

Supported Lipid Bilayer (SLB) and Supported Lipid Monolayer (SLM) Preparation

Carboxylated latex beads (hydrophilic) of 5.7 µm diameter and Divinylbenzene (DVB) coated latex beads (hydrophobic) of diameter 6.4 µm were used for SLB and SLM preparation respectively. To avoid clumping, the beads were sonicated in a bath sonicator for at least 20 minutes. The beads were pelleted at 10,000 g for 5 minutes and then resuspended in milliQ water. For the preparation of SLBs and SLMs, 5 µl of carboxylated and DVB coated latex beads were added respectively with 1µl of 1M NaCl, 69 µl of autoclaved double-distilled water and 20 µl of the BODIPY-C12 labelled liposomes. These were taken together in a siliconized polypropylene microcentrifuge tube and kept for 30 minutes with intermittent vortexing (1 minute vortexing with 5 minutes rest). The mixture was washed thrice by the addition of 1 ml of milliQ water. SLBs and SLMs were pelleted at 10,000 rpm for 5 minutes and then finally resuspended in 200 µl of double-distilled water. The beads (carboxylated and DVB) were normalized to equal numbers using OD_{600} and manual counting using haemocytometer. BODIPY-C12 fluorescence was quantified on SLMs and SLBs by ImageJ to confirm the presence of monolayer and bilayer phospholipid membranes.

Preparation of KIF5B-GFP Enriched Cytosol from HEK-293 Cells

HEK-293T cells were transfected with pCDH-KIF5B-GFP plasmid. Cells were harvested 48 h post transfection, washed with ice-cold PBS, and resuspended in 3 ml of hypotonic buffer (10 mM Hepes [pH 7.4], 1 mM EDTA with protease inhibitor cocktail). The cells were incubated for 20 minutes in ice and centrifuged at 1200 g for 12 minutes. The cell pellet was resuspended in isotonic buffer (250 mM sucrose, 10 mM Hepes [pH 7.4], 1 mM EDTA, protease inhibitor cocktail), followed by lysis in a cell cracker (Isobiotec; 18-micron clearance). The lysate was centrifuged at 1200 g for 12 minutes to remove nuclei and cell debris. The resulting post nuclear supernatant (PNS) was centrifuged at 100,000 g for 1 h at 4^0C and the supernatant (cytosolic fraction) is collected and stored at -80^0C.

Labelling of GST-KTDP with Alexa 647

1 mg of the purified GST-KTDP protein was mixed with 50 ul of 1M sodium bicarbonate solution (pH = 8.5) and 0.5 ul of the Alexa Flour 647 (Invitrogen) dye in dark conditions. The mixture was incubated 2 hours at 4 ºC on nutator followed by dialysis against 1X PBS overnight to remove the unbound dye molecules. Post dialysis, labelled A647-GST-KTDP proteins were collected and concentration was measured using Bradford assay.

KTDP Binding and GFP-Kinesin-1 Removal from SLMs and SLBs

Normalized volumes of SLMs and SLBs were incubated with equal concentration of cytosol containing KIF5B-GFP at 37^0C for 1 h with intermittent mixing (5 sec on/5 sec off) in a thermomixer. KIF5B-GFP bound SLMs and SLBs were separated by centrifugation at 12000g for 10 minutes at room temperature and washed three times with 1X PBS to remove residual cytosol. Collected SLMs and SLBs pellets were resuspended in 1X PBS followed by OD_{600} normalization. Equal volumes of normalized SLMs and SLBs were incubated with 5 µM A647 labelled GST-KTDP at 37^0C for 1 hour with intermittent mixing. The second round of separation was done by centrifugation (12000 g, 10 minutes at RT). The pellets were resuspended in equal volumes of milliQ and imaged in confocal microscope. Fluorescence signals were quantified using ImageJ to compare A647-GST-KTDP binding and KIF5B-GFP removal between SLM and SLB.

Alanine Transaminase (ALT) Activity Test in Cell Culture

To assess hepatocellular toxicity effect of KTDP, McARH-7777 cells transfected with GFP-KTDP and GFP-KTDPmut plasmids were measured for ALT enzyme activity using the ALT Colorimetric Activity Assay Kit (Cayman Chemical Cat# 700260). Briefly, the cells were harvested and lysed to prepare cell lysate using RIPA lysis protocol. After centrifugation at 10,000 g for 15 minutes at 4°C, the supernatant was collected and kept on ice. For the assay 20µl of each sample was mixed with 150 µl of substrate and 20 µl of cofactor, and the plate was incubated at 37°C for 15 minutes. The reaction was initiated by adding 20 µl ALT initiator, and the absorbance was measured at 340 nm once in every 10 minutes up to 60 minutes. The assay and data analysis were done according to manufacturer's instructions and compared across experimental conditions.

Oxygen Consumtion Rate (OCR)

Cellular respiration was measured using Abcam's extracellular oxygen consumption assay kit (ab197243). Cells were seeded in a 96-well plate at a density of 20000 cells/well in 200 µL culture medium and incubated overnight at 37°C in a CO_2 incubator. Cells were transfected with the GFP-KTDP plasmid in four replicate wells, whereas control cells received no plasmid treatment. After 48 hours, oxygen consumption rate was measured following the manufacturer's protocol. The culture medium was replaced with 150 µL fresh medium, followed by the addition of 10 µL reconstituted extracellular O_2 Consumption Reagent. Wells were sealed with 100 µL of pre-warmed high sensitivity mineral oil. Fluorescence was recorded at 2-minute intervals for 120 minutes at excitation/emission wavelengths of 380/650 nm. Fluorescence intensity/lifetime values were plotted against time, and the slope from the linear portion of the curve was calculated to determine the OCR.

Evaluation of LD-Mitochondrial Lipid Flux

McA-RH7777 cells were seeded onto 35-mm glass-bottom confocal dishes and transfected with GFP-KTDP using Lipofectamine 2000 or left untransfected (control). 40 hours post-transfection, cells were subjected to a pulse-chase lipid flux assay (33). Briefly, cells were pulsed with 2 µM BODIPY 558/568 C12 (Red) fluorescent fatty acid analog in the presence of 200 µM oleic acid to facilitate lipid droplet loading and labelling. Following a 16h chase period, cells were washed and mitochondria were labeled with 100 nM MitoTracker Deep Red in Opti-MEM medium for 30 min. Lipid droplets (LDs) were subsequently stained with MDH (50 µM; SM1000a) for 30 min in Opti-MEM. Live-cell imaging was performed in Opti-MEM using an iSIM super-resolution microscope (Nikon) using a 63× oil immersion objective equipped with a stage-top incubation chamber maintained at 37 °C, 5% CO_2, and controlled humidity. Image acquisition, processing, and quantitative analyses were carried out using Nikon imaging software.

Etomoxir Experiments

McA-RH7777 cells were seeded onto acid washed coverslips and transfected with GFP-KTDP using Lipofectamine 2000 as described previously, with untransfected cells serving as controls. Approximately 40h post-transfection, cells were treated with 20 µM etomoxir for 16 h to inhibit carnitine palmitoyltransferase-1 (CPT-I) mediated mitochondrial fatty acid uptake. Following treatment, cells were fixed with 4% paraformaldehyde for 15 min and washed three times with 1× PBS. Lipid droplets were visualized by mounting the cells in mounting medium containing 50 µM monodansylpentane (MDH). Fluorescence images were acquired using a confocal microscope with a 63× oil immersion objective. Image processing and quantitative analysis were performed using ImageJ-Fiji software.

Cellular Morphology Analysis

To evaluate whether KTDP overexpression induces changes in cellular morphology, differential interference contrast (DIC) microscopy was performed using a ZEISS LSM 900 microscope. DIC images were acquired for both GFP-KTDP transfected and untransfected (control) McA-RH7777 cells. A total of 25 cells from each condition were randomly selected for analysis. Cell boundaries were manually delineated using ImageJ-Fiji software, and the corresponding cell surface areas were calculated. The quantified surface areas were subsequently compared between KTDP-overexpressing and control cells.

Measurement of TG and Cholesterol Using Commercial Assay Kit and LC-MS

For measurement of secreted and cellular TG, both KTDP and KTDP-mut plasmids were transfected into McARH-7777 cells using the protocol explained earlier. 48 hours post transfection, cells were treated with 0.4 mM OA conjugated with BSA in incomplete media (no FBS) for 6 hours followed by washing with phenol-red-free incomplete media containing 0.5% fatty acid free BSA. For chase period, the cells were further cultured in phenol-red-free incomplete media with 0.5% fatty acid free BSA for 4 h. At the end of the chase period the media were collected and cells were harvested and stored in -80^{0}C. Cell pellets were rinsed with cold 1X PBS and resuspended in RIPA lysis buffer supplemented with protease inhibitors. Cells were incubated on ice for 30 minutes with vortexing briefly in every 5-10 minutes followed by centrifugation at 14000g for 15 minutes at 4^{0}C. The supernatant containing the cell lysate is collected. TG and total cholesterol measurement were conducted in secreted media and cell lysate using colorimetric assay kits (Elabscience) following the manufacturer's protocol.

Secreted media and cell pellets were used for quantitative LC-MS analysis to measure TG, cholesterol and free fatty acids (FFA) following the protocol previously described in (22). Briefly, media and cell pellets (4 biological replicate for each group) were resuspended in 1 mL 1X-Phosphate buffered saline (PBS) and made up to a 4 mL mixture of 2:1:1 chloroform ($CHCl_3$):methanol (MeOH): PBS. For semi-quantitative analysis of lipids, 1 nmol of an unnatural monoacylglycerol (C15:0 MAG) was added as internal standard for positive mode analytes. This homogenate mixture was vigorously vortexed and centrifuged at 3000g for 10 minutes to separate the mixture into an organic phase (bottom) and an aqueous phase (top) separated by a protein disk. The organic phase was removed by pipetting and stored on ice. To enhance the extraction of phospholipids from the aqueous layer, 100 µL of formic acid (MS grade, Honeywell, Catalog # 94318) was added, and this mixture was vigorously mixed. 2 mL of $CHCl_3$ was added, and this mixture was vortexed and centrifuged as described previously. The organic layer was pooled with the one from the first extraction step and dried under a stream of nitrogen gas. The dried lipid extracts were re-solubilized in 200 µL of 2:1 $CHCl_3$:MeOH and 10 µL was injected into an Agilent 6545 LC-QTOF (quadrupole-time-of-flight) LC-MS/MS for semiquantitative analysis using high-resolution auto MS-MS methods and chromatography techniques. LC separation employed a Gemini 5U C-18 column (Phenomenex) coupled with a Gemini guard column (Phenomenex, 4x3 mm, Phenomenex security cartridge). The buffers for positive ion mode runs consisted of 95:5 H_2O: MeOH + 0.1 % Formic acid + 10 mM ammonium formate (buffer A) and 60:35:5 Isopropanol: MeOH: H_2O + 0.1% Formic acid + 10 mM ammonium formate hydroxide (buffer B). Methods spanned 60 minutes, starting with 0.3 mL/min 100% buffer A for 5 minutes, 0.5 mL/min linear gradient to 100% buffer B over 40 minutes, 0.5 mL/min 100% buffer B for 10 minutes, and equilibration with 0.5 mL/min 100% buffer A for 5 minutes. ESI-MS analysis settings included drying gas and sheath gas temperatures at 320 °C, a flow rate of 10 L/min for both drying gas and sheath gas respectively, a fragmenting voltage of 150V, capillary voltage of 4 kV, nebulizer (ion source gas) pressure set at 45 psi, and nozzle voltage of 1 kV. For analysis, a lipid library in the form of a Personal Compound Database Library (PCDL) was employed, and peak validation relied on relative retention times and fragments acquired. Quantification of all lipid species involved the normalization of areas under the curve to the corresponding internal standard area and further normalization to the total cell count. Subsequently, the changes were plotted for each lipid in comparison to the appropriate control within the group.

Cholesterol and cholesteryl esters were quantified using an Agilent 6545 QTOF mass spectrometer coupled with a 1290 Infinity II UHPLC system *(See Ref. 2 in SI Reference List)*. Separation was performed on a Gemini C18 column using a 30-minute gradient LC method with formic acid and ammonium formate-containing solvents. MS acquisition was done in positive ion mode using Auto-MS/MS with optimized parameters and a preferred ion list targeting cholesterol and cholesteryl esters.

Preparation and Administration of Egg Liposomes to Zebrafish Larvae

Liposome preparation and feeding procedures were conducted following (35), with slight modifications. To prepare BODIPY-tagged liposomes, red fluorescently labelled BODIPY™ FL C12 (Invitrogen, Cat. No. D3822) was evaporated under a stream of nitrogen (N_2) and resuspended in 10 µL of 100% ethanol in Eppendorf tubes. Zebrafish embryo medium (EM; 90 µL) was then added to the resuspended solution. The resulting fluorescent stock solutions were protected from light and stored at 4°C.

For the liposome feeding solution, 1 mL of frozen chicken egg yolk (stored at -80°C) was thawed to room temperature and mixed with 19 mL of zebrafish EM. The peptides KTDP and KTDP-Mut were incorporated at this stage, ensuring a final concentration of 250 µg/mL of peptide in the feeding medium. The mixture was pulse sonicated for 40 s (1s on, 1s off; output intensity: 40%), filtered through a 40 µm cell strainer, and subjected to a second sonication step before collection. The resuspended BODIPY C12 was then added to 5 mL of the emulsion and vortexed for 30 seconds to achieve a final concentration of 6.4 µM BODIPY C12.

Freshly prepared liposomes were diluted tenfold and introduced into a flow chamber on a transparent glass slide. After allowing the liposomes to settle for 5 minutes to facilitate adherence to the slide's bottom surface, imaging was performed using a confocal microscope (Zeiss LSM 900) equipped with a 63X objective. ESID and GFP excitation lasers were utilized to visualize liposome particles, confirming both the successful formation of liposomes and the encapsulation of KTDP.

Prior to liposome feeding, larvae were screened for any developmental abnormalities. A total of 15 healthy larvae per well were placed in 3 mL of the liposome solution and fed in a 6-well culture dish on an orbital shaker (30 rpm) for 6 hours across three experimental groups: BODIPY Liposomes (Empty), BODIPY-liposomes with KTD Wild type-GFP peptide (KTDP), and BODIPY-liposomes with KTD mutant-GFP peptide (KTDP-Mut). No observable signs of toxicity were detected during and after the feeding. Successful feeding was confirmed by the presence of red fluorescence in the gut area, observed under a fluorescence microscope (Olympus MVX10). To assess the delivery of the peptides specifically to the liver, a separate set of experiments was conducted using the same three experimental groups (without BODIPY-tagged liposomes) on transgenic zebrafish larvae [Tg(fabp10a:DsRed)], where liver cells express red fluorescence under the fabp10a promoter. After feeding in both sets of experiments, larvae underwent three sequential washes in fresh EM (5 minutes per wash) to remove residual liposomes or free peptides, followed by anesthesia using Tricaine. Larvae were then fixed in 4% paraformaldehyde for imaging or snap-frozen in liquid nitrogen and stored at -80°C for biochemical assays.

Efficiency of Liposome-mediated Peptide Delivery in Zebrafish Larvae

To evaluate the efficiency of liposomal delivery for GFP-tagged peptides (KTDP and KTDP-Mut), a standard curve was generated by plotting protein concentration against GFP fluorescence intensity. GFP excitation and emission wavelengths were set at 488 nm and 509 nm, respectively, with fluorescence measurements conducted using a multimode plate reader (Agilent Synergy H1) in a 96-well plate. Serial dilutions of KTDP-GFP were prepared at concentrations of 40, 50, 67, 100, 125, 200, and 250 µg/mL, and their corresponding fluorescence intensities were recorded. The resulting standard curve was used for quantification. Following peptide administration, 6 dpf zebrafish larvae were incubated with treatments for 6 hours in 6-well plates, as previously described. 50 Larvae were distributed per condition, covering three experimental groups: liposomes without GFP-KTDP, GFP-KTDP (250 µg/mL) encapsulated in liposomes, and GFP-KTDP (250 µg/mL) directly mixed into the media without liposomes. Larvae were homogenized using probe sonication (2s on, 3s off; total 40s; 40% intensity), followed by centrifugation at 10,000 g for 10 minutes to collect the resulting supernatants. To account for background fluorescence, lysates from the group containing liposomes without GFP-KTDP were used as the negative control. The fluorescence intensity obtained from these samples was subtracted from all experimental groups to ensure accurate assessment of liposomal delivery efficiency.

Lipid extraction and Estimation of TG and Total Cholesterol in Larval Zebrafish

For the quantification of TG and total cholesterol, frozen zebrafish larvae were thawed on ice and bisected using a blade to separate the head region (containing visceral organs like liver, gut, brain etc.) from the tail region (primarily vasculature). The head and tail portions from 60 larvae were pooled to constitute one biological replicate, and this entire procedure was performed in triplicate (n=3). The pooled head tissues and the pooled tail tissues from the same 60 larvae (for one replicate) were each placed in 180 µL of PBS and sonicated using a pulse sonicator (2s on, 3s off; 50% intensity) for 30 seconds. The resulting homogenates were then centrifuged at 12,000 × g for 10 minutes at 4°C, and the supernatants were collected separately for head and tail regions.

Lipids were extracted from the supernatants of both head and tail homogenates using the chloroform-methanol method. Briefly, the collected supernatants were completely dried in a vacuum concentrator under constant rotation at room temperature. Once dried, lipids were extracted by adding a chloroform-methanol (2:1) mixture to the dried supernatant, followed by centrifugation at 12,000 × g for 15 minutes. The organic phase containing chloroform was collected and dried under a nitrogen (N_2) stream. The dried lipids were then reconstituted in 50 µL of isopropanol and stored at -20°C until further use. Protein concentration was determined using Bradford's method from the initial aqueous supernatants of both head and tail homogenates for each replicate. Prior to TAG and total cholesterol measurements, the resuspended lipid extracts were normalized for protein content. The same tail lysates were subjected to lipid extraction and lipidomics analysis as described above.

TG and total cholesterol quantification was performed using colorimetric assay kits (Elabscience, Cat No. E-BC-K238 and E-BC-K109-S respectively) following the manufacturer's protocol. Briefly, the protein-normalized lipid extracts were mixed with the respective kit's reagent, incubated at 37°C for 30 minutes, and the absorbance was measured at 510 nm using a microplate reader (Agilent Synergy H1).

Imaging and Quantification of Circulatory Lipids in Zebrafish Larvae

Zebrafish embryos, following experimental treatments, were fixed in 4% paraformaldehyde (PFA) overnight at 4°C. To remove residual fixative, the embryos were washed three times with phosphate-buffered saline (PBS). For enhanced optical clarity and preservation prior to imaging, the fixed embryos were transferred to a 1:1 PBS:Glycerol solution and incubated overnight at 4°C. Subsequently, embryos were mounted on glass slides using glycerol-based mounting media (Glycerol + DABCO), covered with a 10 mm round glass coverslip, and the edges were sealed with transparent nail polish to prevent dehydration. Slides were stored at 4°C overnight to allow complete drying. Imaging was primarily performed using a confocal microscope (Zeiss, LSM 900). Fluorescence intensity quantification was carried out using ImageJ software. For specific visualization of lipid droplets (LDs) within the larval liver, a 63x oil immersion objective was used, and fluorescence intensities were measured from random regions of interest (n=6) across the entire liver. To quantify LDs, the number of LDs per cell was counted from randomly selected cells (n=10) across the liver using ImageJ. These counts were then plotted for analysis.

To monitor the long-term distribution of lipids in the circulation, particularly within the tail vasculature of zebrafish larvae, Nile Red staining was performed. Six randomly selected larvae from each treatment group at various time points post incubation (6h, 12h, 24h, 48h, 120h) were fixed in 4% PFA overnight at 4°C and washed three times in PBS. Nile Red staining was conducted

following established methods *(See Ref. 3 in SI Reference List)*, where larvae were immersed in a 0.79 mM Nile Red solution in embryo media and incubated in the dark at room temperature for 30 minutes. Excess dye was removed by three washes in PBS, and the stained larvae were then transferred to 50% glycerol in PBS and stored overnight at 4°C. Finally, larvae were mounted on transparent glass slides in 70% glycerol with DABCO for imaging. Confocal microscopy for Nile Red imaging was performed using the Zeiss confocal microscope (LSM 900) with excitation at 543 nm and emission at 598 nm. Consistent imaging parameters (laser intensity and exposure time) were maintained across all samples. Nile Red fluorescence intensity in the tail vasculature, indicative of circulating lipids, was quantified using ImageJ software and expressed as fold change relative to larvae treated with empty liposomes at each corresponding time point.

Effect of KTDP on Morphology, Mortality and Locomotion of Zebrafish Larvae

To evaluate the impact of KTDP on zebrafish larvae, larvae were subjected to different liposome treatments. For each experimental condition, zebrafish larvae (n=30) were placed in each well of a 6-well plate, with 3 ml of media added to each well. All experiments were performed in triplicate to ensure reproducibility. Larvae were incubated for 6 hours (at 30 RPM shaking, 28.5 ℃) with either empty egg liposomes (vehicle control), liposomes encapsulating KTDP, or liposomes encapsulating a KTDP-Mutant peptide, prepared as previously described. Following this 6-hour incubation period, larvae were transferred to fresh E3 medium and maintained under standard conditions with normal feeding protocols.

Morphological evaluations were conducted at 6, 12, 24, 48 and 120 hours post-incubation (hpi) with the liposome treatments. Prior to imaging, larvae were anesthetized in E3 medium containing 0.06% Tricaine. Brightfield images were captured using an stereomicroscope (Olympus MVX10). Consistent illumination, magnification, and exposure settings were maintained across all samples and time points. Qualitative assessments focused on gross morphological changes, including body curvature, edema formation (e.g., pericardial), developmental milestones (e.g., swim bladder inflation, yolk sac absorption), and overall structural integrity compared to the vehicle control group. Survival rates were monitored daily for 120 hours post-incubation. Larvae were examined at 24-hour intervals, and mortality was recorded. Cumulative mortality percentages were calculated for each group at each time point. Survival curves were generated based on these data. Environmental conditions (28°C, 14:10 light:dark cycle) were strictly maintained throughout the assay period.

Escape response was assessed at 120 hpi as described with some modifications *(See Ref. 4 in SI Reference List)*. Individual larvae (n=10 per condition) were transferred to the center (Zone 0) of a 35 mm Petri dish containing E3 medium. The dish surface was conceptually divided into concentric zones (Diameter Zone 0: 7.78 mm, Zone 1: 16.85 mm, Zone 2: 25.92 mm, Zone 3: 35 mm). A standardized tactile stimulus was delivered to the caudal fin region using a fine micropipette tip to elicit an escape response. The zone in which the larva first ceased significant movement following the stimulus was recorded. The distribution of larvae across zones was plotted as the mean of their zone no. Spontaneous locomotor activity was assessed at 120 hours post-incubation (hpi). Briefly, individual larvae were placed in separate wells of a 6-well plate containing E3 medium. Larval movement was recorded for 3 minutes using a video camera (30 fps) mounted above an LED-illuminated surface supporting the plate. Video files were processed using ZebraZoom software to automatically track larval position and quantify locomotion parameters *(See Ref. 5 in SI Reference List)*. For comparative analysis, the mean total distance traveled (mm) and mean angular velocity (deg/s) for each treatment group were expressed as fold changes relative to the mean values obtained from the vehicle control group (empty liposomes). All behavioral recordings were conducted at 28°C under consistent lighting conditions to minimize external variability.

Animal Husbandry and Grouping for Adult Zebrafish Experiments

One-year-old adult zebrafish (*Danio Rerio*) of both sexes were equally and randomly allocated into two experimental groups: (1) Empty liposomes, and (2) KTDP-liposomes. Fish were housed in a custom-designed standalone recirculating aquaculture system. This system consisted of fish tanks placed on a draining tray connected to a lower reservoir with biological filters and a submersible pump. Water was continuously cycled back into the tanks via inlet tubing, and overflow was drained back to the reservoir, maintaining stable water quality and flow conditions. Water quality parameters such as temperature, pH, and ammonia levels were monitored regularly to ensure optimal husbandry conditions.

KTDP Administration via Oral Gavage in Adult Zebrafish

KTDP was encapsulated in egg-yolk liposomes and administered at a concentration of 250 µg/mL, identical to the dose used in larval studies. Each fish received 5 µL of liposomal solution once daily in the morning, followed by standard feeding for the rest of the day. Gavage was performed for three consecutive days.

Due to the technical challenges associated with oral gavaging in adult zebrafish, we adopted and modified the method described by Collymore (37). Briefly, a custom sponge block with a midline groove was used to immobilize the fish in a vertical orientation. A femtotip, trimmed to allow better flow, was fitted onto a 10 µL pipette and loaded with the liposomal dose. Fish were anesthetized in 100 µg/mL tricaine, placed in the moistened sponge with their head exposed, and the tip was gently inserted ~3 cm into the oral cavity to dispense the solution. To prevent regurgitation, fish were kept vertically in the sponge for an additional 30 seconds post-gavage before being transferred to a recovery tank for 15 minutes. Any fish displaying bleeding, distress, or abnormal behavior were excluded from further experimentation.

Sample Collection from Adult Zebrafish

On the third day, after the final dosing, fish were fasted and sacrificed four hours post-gavage. Blood was collected from the tail vein. Following anesthesia, fish were wiped with 70% ethanol, and the tail was severed just caudal to the anal fin using a sterile blade. Fish were then placed in a perforated 0.5 mL microcentrifuge tube, which was inserted into a 1.5 mL collection tube and centrifuged at 50 × g to collect blood. Serum was separated from the supernatant for further analysis. Liver and anterior gut tissues

were dissected, washed in cold PBS, and imaged immediately under an Olympus stereomicroscope in FITC and TRITC channels. Tissues were then snap-frozen in liquid nitrogen and stored at −20°C for further biochemical analysis. Total cholesterol and TG in serum and liver lysates were quantified using commercially available colorimetric assay kits, as described previously. Serum samples collected from fish gavaged with empty liposomes, or liposomes containing KTDP or KTDP-mut peptide were subjected to liver function testing at a pathology laboratory to determine serum aspartate aminotransferase (AST) and alanine aminotransferase (ALT) levels. The AST/ALT ratio was calculated and compared across experimental groups

Molecular Dynamics Simulations

System setup

The initial PDB structure of the Kinesin Tail Domain protein (KTDP) is modeled in I-TASSER *(See Ref. 6 in SI Reference List)* due to the unavailability of experimentally reported structures in the Protein Data Bank. The modeled structure consisting of 110 residues, is solvated in explicit water and equilibrated for 1 ns in an isothermal-isobaric (NPT) ensemble. The final equilibrated protein structure is extracted for further modeling steps. Two membrane-protein systems are prepared to study the differential binding of KTDP with monolayer and bilayer cargo. A model monolayer system is constructed by extracting the PDB structures of 1,2-dioleoyl-sn-glycero-3-phosphocholine (DOPC), 1,2-dioleoyl-sn-glycero-3-phosphate (DOPA), and Triacylglycerols (TG) molecules from membrane patches built in CHARMM-GUI *(See Ref. 7 and 8 in SI Reference List)*. PACKMOL *(See Ref. 9 in SI Reference List)* was used to build the model monolayer system by defining three boxes at different square planes with of sides measuring ca. 12.3 nm. The planar surfaces are designated to the membrane leaflets consisting of DOPC and DOPA (first and third) in a 95:5 ratio and a neutral lipid layer of TGs (second). The upper and lower leaflet consisting of DOPC and DOPA is added to support the middle TG layer without compromising the atomic resolution and stability. PACKMOL-generated monolayer PDB structure is solvated with the TIP3P water model and equilibrated for 400 ns at 303 K in the isothermal-isobaric (NPT) ensemble. The final PDB structure is taken as the initial monolayer configuration interacting with the KTDP. The bilayer system (devoid of TG) is generated within the CHARMM-GUI server. The center of mass (COM) of KTDP is kept ca. 2.5 nm apart from the COM of phosphate of upper leaflet in both systems to ensure unbiased interaction. Two 6.4 nm thick water layers are introduced at both sides of the leaflets in the monolayer, whereas in the bilayer, the water thickness is 5 nm at each side. The final monolayer and bilayer consisted of 299888 and 196670 atoms, respectively. The number of molecules in monolayer and bilayer systems are :-

Monolayer DOPC - 418 (209 in each leaflet), DOPA - 22 (11 in each leaflet), TG - 300, Water - 62577, Ions - 9 (Na+ to neutralize the system).
Bilayer DOPC - 418 (209 in each leaflet), DOPA - 22 (11 in each leaflet), Water - 44871, Ions - 9 (Na+ to neutralize the system).

Fully atomistic molecular dynamics (MD) simulations are performed with the GROMACS 21.1 MD engine *(See Ref. 10 in SI Reference List)* with the CHARMM36m forcefield *(See Ref. 11 in SI Reference List)*. TIP3P explicit water model is used for solvation. Bond lengths involving hydrogen atoms are held fixed using the LINCS algorithm *(See Ref. 12 in SI Reference List)*. A non-bonded cutoff of 1.2 nm is used, and long-range electrostatic interactions are computed with the Particle Mesh Ewald (PME) method *(See Ref. 13 in SI Reference List)*. The systems are initially minimized with the steepest descent algorithm, The system is equilibrated for 5 ns at a constant temperature of 310 K using the Nose-Hoover thermostat and an isobaric and semi-isotopic pressure of 1 bar using the Parrinello-Rahman barostat. Velocities are periodically reassigned over 1 µs, followed by production runs of 0.25 µs. Effective trajectories of 1.25 µs were generated for each system with a timestep of 2.0 fs. ChimeraX is used for visualization *(See Ref. 14 in SI Reference List)*.

Analysis Area per lipid (APL) is defined as the accessible leaflet area per lipid molecule. A higher APL value represents a higher accessible area for a lipid, corresponding to poorer lipid packing *(See Ref. 15,16 in SI Reference List)*. The standardized FATSLiM algorithm was used for the APL *(See Ref. 17 in SI Reference List)*.

Lipid occupancy (O_{ij}, where i = lipid type ; j = peptide residue) is defined by the time fraction in which a particular amino acid residue forms contact with a lipid molecule, and is estimated with a cutoff distance of 0.7 nm. This calculation is done by using the PyLipID toolkit. For the ith lipid (DOPC or DOPA) and j^{th} peptide residue, the ΔO_{ij} is calculated as the following,

$$\Delta O_{ij} = O_{ij}^{monolayer} - O_{ij}^{bilayer}$$

The average DOPC-DOPA occupancy of a KTDP residue (= $\langle O \rangle_j$) is determined by averaging the DOPC and DOPA lipid occupancies of each KTDP residue for monolayer and bilayer systems.

Lipid fraction per residue (L_f) is determined as $L_f = \frac{1}{N} * L_n$. Here N is the number of residues interacting with Ln lipids within a cutoff of 0.7 nm. Interactions with the headgroups (for DOPC and DOPA) or with TG molecules were considered. of each residue having favorable interaction in monolayer and bilayer. The smooth probability distribution envelope $P(L_f)$ was obtained from the discrete data with Kernel Density Estimation (KDE).

REFERENCE LIST IN SUPPORTING INFORMATION, *Tripathy et. al.*

1) O. L. Knittelfelder, S. D. Kohlwein, Thin-layer chromatography to separate phospholipids and neutral lipids from 836 yeast. Cold Spring Harb. Protoc. 2017, 412–415 (2017).
2) A. Chandramouli, S. S. Kamat, A facile LC-MS method for profiling cholesterol and cholesteryl esters in mammalian cells and tissues. Biochemistry 63, 839 (2024), 10.1021/ACS.BIOCHEM.4C00160.
3) K. Balamurugan et al., Protocol to evaluate hyperlipidemia in zebrafish larvae. STAR Protoc. 3, 840 (2022), 10.1016/j.xpro.2022.101819.
4) L. Guzman et al., Evaluation of the effects of acetylcholinesterase inhibitors in the zebrafish touch-evoked 842 response: Quantitative vs. qualitative assessment. Environ. Sci. Europe 32, 145 (2020).
5) O. Mirat, J. R. Sternberg, K. E. Severi, C. Wyart, ZebraZoom: An automated program for high-throughput behavioral 844 analysis and categorization. Front. Neural Circuits 7, 845 (2013), 10.3389/FNCIR.2013.00107.
6) J. Yang et al., The I-TASSER suite: Protein structure and function prediction. Nat. Methods 12, 7–8 (2014).
7) J. Lee et al., CHARMM-GUI input generator for NAMD, GROMACS, AMBER, OpenMM, and 847 CHARMM/OpenMM simulations using the CHARMM36 additive force field. J. Chem. Theory Comput. 12, 405–413 (2016).
8) S. Jo, T. Kim, V. G. Iyer, W. Im, CHARMM-GUI: A web-based graphical user interface for CHARMM. J. Comput. Chem. 29, 1859–1865 (2008).
9) L. Martinez, R. Andrade, E. G. Birgin, J. M. Martínez, PACKMOL: A package for building initial configurations for 852 molecular dynamics simulations. J. Comput. Chem. 30, 2157–2164 (2009).
10) J. Lindahl, T. Abraham, B. Hess, D. van der Spoel, GROMACS 2021.1 Manual 2021.1 Manual. 10.5281/ZENODO.4561625. 854 (2021).
11) J. Huang et al., CHARMM36m: An improved force field for folded and intrinsically disordered proteins. Nat. Methods 14, 71–73 (2016).
12) B. Hess, H. Bekker, H. J. C. Berendsen, J. G. E. M. Fraaije, LINCS: A linear constraint solver for molecular simulations. J. Comput. Chem. 18, 1463–1472 (1997).
13) H. G. Petersen, Accuracy and efficiency of the particle mesh Ewald method. J. Chem. Phys. 859, 3668–3679 (1995).
14) E. F. Pettersen et al., UCSF ChimeraX: Structure visualization for researchers, educators, and developers. Science 30, 70–82 (2021).
15) E. Falck, M. Patra, M. Karttunen, M. T. Hyvönen, I. Vattulainen, Lessons of slicing membranes: Interplay of packing, 863 free area, and lateral diffusion in phospholipid/cholesterol bilayers. Biophys. J. 87, 1076–1091 (2004).
16) P. F. F. Almeida, T. E. Thompson, Lateral diffusion in the liquid phases of 865 dimyristoylphosphatidylcholine/cholesterol lipid bilayers: A free volume analysis. Biochemistry 31, 6739–6747 (1992).
17) S. Buchoux, FATSLiM: A fast and robust software to analyze MD simulations of membranes. Bioinformatics 33, 133–134 (2017).

SUPPLEMENTARY MOVIE CAPTIONS, *Tripathy et. al.*

SUPPLEMENTARY MOVIE 1

Video showing the interaction of Kinesin tail domain peptide (KTDP) with Bilayer Membrane over a period of 125 microseconds. This video is based on MD simulations (see main text).

SUPPLEMENTARY MOVIE 2

Video showing the interaction of Kinesin tail domain peptide (KTDP) with Monolayer Membrane over a period of 125 microseconds. This video is based on MD simulations (see main text).
